# Development of a three-dimensional scoring model for the assessment of continuous glucose monitoring data in type 1 diabetes

**DOI:** 10.1136/bmjdrc-2024-004350

**Published:** 2024-09-05

**Authors:** Jeanie Dawnbringer, Henrik Hill, Markus Lundgren, Sergiu-Bogdan Catrina, José Caballero-Corbalan, Lars Cederblad, Per-Ola Carlsson, Daniel Espes

**Affiliations:** 1 OneTwo Analytics AB, Stockholm, Sweden; 2 Department of Women’s and Children’s Health, Uppsala University, Uppsala, Sweden; 3 Department of Clinical Sciences Malmö, Lund University, Malmö, Sweden; 4 Department of Pediatrics, Kristianstad Hospital, Kristianstad, Sweden; 5 Department of Molecular Medicine and Surgery, Karolinska Institutet, Stockholm, Sweden; 6 Center for Diabetes, Academic specialist Center, Stockholm, Sweden; 7 Department of Medical Sciences, Uppsala University, Uppsala, Sweden; 8 Department of Medical Cell Biology, Uppsala University, Uppsala, Sweden; 9 Science for Life Laboratory, Department of Medical Sciences, Uppsala University, Uppsala, Sweden; 10 Science for Life Laboratory, Department of Medical Cell Biology, Uppsala University, Uppsala, Sweden

**Keywords:** Continuous Glucose Monitoring, Hypoglycemia, Hyperglycemia, Population Health

## Abstract

**Introduction:**

Despite the improvements in diabetes management by continuous glucose monitoring (CGM) it is difficult to capture the complexity of CGM data in one metric. We aimed to develop a clinically relevant multidimensional scoring model with the capacity to identify the most alarming CGM episodes and/or patients from a large cohort.

**Research design and methods:**

Retrospective CGM data from 2017 to 2020 available in electronic medical records were collected from n=613 individuals with type 1 diabetes (total 82 114 days). A scoring model was developed based on three metrics; glycemic variability percentage, low blood glucose index and high blood glucose index. Values for each dimension were normalized to a numeric score between 0–100. To identify the most representative score for an extended time period, multiple ways to combine the mean score of each dimension were evaluated. Correlations of the scoring model with CGM metrics were computed. The scoring model was compared with interpretations of a clinical expert board (CEB).

**Results:**

The dimension of hypoglycemia must be weighted to be representative, whereas the other two can be represented by their overall mean. The scoring model correlated well with established CGM metrics. Applying a score of ≥80 as the cut-off for identifying time periods with a ‘true’ target fulfillment (ie, reaching all targets for CGM metrics) resulted in an accuracy of 93.4% and a specificity of 97.1%. The accuracy of the scoring model when compared with the CEB was high for identifying the most alarming CGM curves within each dimension of glucose control (overall 86.5%).

**Conclusions:**

Our scoring model captures the complexity of CGM data and can identify both the most alarming dimension of glycemia and the individuals in most urgent need of assistance. This could become a valuable tool for population management at diabetes clinics to enable healthcare providers to stratify care to the patients in greatest need of clinical attention.

**WHAT IS ALREADY KNOWN ON THIS TOPIC**

- Continuous glucose monitoring (CGM) has become an invaluable tool in diabetes management but the amount of data constitutes a great challenge.
- Most of the standard CGM metrics are one-dimensional and do, therefore, not alone capture the complexity of glycemia.
- We have tested the hypothesis that a multidimensional scoring model could reflect the complexity of glycemia and be a useful tool for prioritizing patients in need of more urgent clinical attention.

**WHAT THIS STUDY ADDS**

- We have established a three-dimensional scoring model which takes into account hypoglycemia, hyperglycemia and glucose variability.
- Our scoring model correlates well with standard CGM metrics.
- The developed scoring model also correlates well with the interpretation of a clinical expert board.

**HOW THIS STUDY MIGHT AFFECT RESEARCH, PRACTICE OR POLICY**

- A multidimensional scoring model for interpretation of CGM data can be a valuable tool for prioritizing patients in more urgent need of clinical attention and an important metric to track longitudinally in order to detect alarming changes of glycemia.

## Introduction

 Continuous glucose monitoring (CGM) has dramatically improved the possibilities to map glucose fluctuations and has become an invaluable tool for people living with type 1 diabetes (T1D). For people living with T1D the current glucose level and trend for the nearest future is of most interest for all the decision that has to be made regarding insulin therapy and dosing in their daily lives.^1^ The healthcare sector, on the other hand, is more interested in retrospective data in order to evaluate mean values in relation to treatment targets and to identify trends that can aid the decision on how to adjust the therapy.^2^ Traditionally hemoglobin A1c (HbA1c) has been the gold standard for assessing glycemic targets and is still the most well studied end point in terms of future risk for complications.^3^,^5^ Although HbA1c is an important measure, it also has a number of shortcomings, the most apparent being its inability to reflect glucose variability and the frequency of hypoglycemic events. Since the introduction of CGM and the ambulatory glucose profile (AGP), a number of additional metrics are now used for assessing glycemic targets. There are clinical consensus statements based on AGP metrics regarding treatment targets for CGM data in both T1D and type 2 diabetes.^2^ However, it is still a challenge to evaluate the current status of glycemic targets in a time-efficient manner since most AGP metrics are one-dimensional and the same patient may fulfil one of the parameters while failing to meet others. Based on these challenges different composite metrics have been proposed, as reviewed, for instance, by Nguyen *et al* in 2020.^6^ In general, most composite metrics or scoring models are optimized to either identify the risk for hypoglycemia/hyperglycemia or to provide an overall estimate of glucose control. However, in recent years glucose variability and its implications, both for the risk of complications and impact on quality of life, have gradually started to become more accepted.^7 8^ There are several metrics used to describe glucose variability^9^ out of which the coefficient of variation (CV%) is currently the one adopted by consensus guidelines.^2^ However, CV% does not take into account the frequency of glucose variability and can therefore, for instance, not identify patients with frequent excursions of glucose within the time in range (TIR, 3.9–10 mmol/L). Glycemic variability percentage (GVP) on the other hand accounts for both the amplitude and frequency of glucose excursions and can also discriminate glucose curves of individuals without diabetes from those with T1D and, in addition, grade the level of variability of patients with T1D.^10^ There are several metrics which have been designed to capture the risk of both hypoglycemia and hyperglycemia. Ideally such a metric would take into account the difference in frequency and duration of hypoglycemia and the more acute implications of such events when compared with hyperglycemia. In fact, studies have shown that 6%–10% of all deaths among patients with T1D are caused by hypoglycemia.^5 11 12^ The risk for severe hypoglycemia is captured by the low blood glucose index (LBGI) which has adopted a penalty function designed to penalize low glucose values on a steeper curve.^13^ Conversely, there is also a high blood glucose index (HBGI) which consists of a penalty function for increasing glucose levels which obviously is also of great importance since the long-term complications due to hyperglycemia contribute to the reduction of life expectancy in T1D of almost 10 years.^14 15^

We aimed to develop a clinically relevant multidimensional score taking into account both glucose variability, hypoglycemia and hyperglycemia. Based on the currently published metrics for assessing CGM data we developed a scoring model based on LBGI, HBGI and GVP in order to also incorporate a sensitive measure of glucose variability. Our aim was to develop a scoring model that could both identify the most alarming dimension of glucose control and provide a single normalized numeric value representative thereof.

## Research design and methods

### Data collection and preparation

The presented study was based on retrospective CGM and clinical data collected at the Department of Endocrinology and Diabetology and the Department of Pediatric Endocrinology and Diabetology at Uppsala University Hospital, Sweden. Retrospective CGM data from the years 2017–2020 were collected from n=613 patients (n=378 adults and n=235 children/youths) with T1D all of whom were under follow-up at Uppsala University Hospital under standard care for diabetes management with free access to CGM sensors and received intensive insulin treatment. The CGM data were collected from electronic medical records based on availability. Since the data set contained data from different types of CGM sensors, the time interval was downsampled to 15 min in order to harmonize the data. Days with a data availability <70% were excluded from analysis resulting in a mean of 134±60 days of CGM data per patient (in total 82 114 patient days). These data were used to evaluate and normalize data for the three-dimensional scoring model. A separate data set (ie, not included in the normalization) was collected from n=20 adults and n=20 children/adolescents with T1D and prepared as described above and used for the clinical expert board (CEB) validation.

### Generation of a multidimensional score

To capture the complexity of CGM data, a scoring model was constructed based on three dimensions of glucose control, namely (1) Hypoglycemia, (2) Hyperglycemia and (3) Variability. For the aspect of risk associated with hyperglycemia and hypoglycemia, there are several metrics available including, of course, the raw glucose levels. However, to capture the severity of hypoglycemia, considering also the often shorter duration, there is a need for weighting of the hypoglycemic and hyperglycemic events. Based on previously published data, we therefore adopted the LBGI/HBGI, which uses a penalty function for both hypoglycemia and hyperglycemia.^13^ Briefly described, both LBGI and HBGI are formulas that compute a risk index ranging from 0 to 100, respectively, for hypoglycemic and hyperglycemic events. For variability, there are several metrics available, but in order to capture both the amplitude and frequency of glucose fluctuations, the GVP was incorporated.^10^ For each individual, a daily LBGI, HBGI and GVP was computed. By applying a min-max normalization for each of the variables a daily score between 0 and 100 was computed. For LBGI and HBGI the minimum value of the respective indices (ie, 0) was applied which resulted in a score of 100 for that respective dimension in our model. For GVP a value of 20% was applied as the lowest (*ie, ‘best’*) possible level based on the published literature.^10^ Hence, a GVP of 20% will result in a variability score of 100 in our model. For the maximum values, the 98th percentiles from the data set (consisting of 82 114 days of CGM data derived from n=613 patients with T1D) were used to reflect the score of 0 (ie, the most alarming) for all three dimensions. For the HBGI normalization, the maximum values were separated for adults and children/adolescents due to the differences in treatment targets (HbA1c 52 mmol/mol vs 48 mmol/mol). The minimum and maximum values of LBGI, HBGI and GVP values are presented in table 1.

**Table 1:** Normalization values used to achieve scores between 0 and 100 for the three dimensions hypoglycemia, hyperglycemia and variability

	Minimum	Maximum	
LBGI	0	8	
HBGI	0	Adult	Pediatric
		32	30
GVP	20	67	

GVP, glycemic variability percentage; HBGI, High Blood Glucose Index; LBGI, Low Blood Glucose Index.

### Selecting a single representative score based on the three dimensions

To present a single score that is representative for a longer time period, we examined multiple ways to combine the daily scores of each dimension. The goal was to identify the most representative score while still maintaining a high sensitivity to detect an increased risk in a single dimension regardless of the other two. Different combinations were evaluated based on the correlations of the single score with the following AGP metrics:

- Time above range (TAR, > 10.0 mmol/L)
- Time severe above range (TSAR, > 13.9 mmol/L)
- Time below range (TBR, < 3.9 mmol/L)
- Time severe below range (TSBR, < 3.0 mmol/L)
- CV%

The most straightforward approach would be to calculate the mean of all daily scores for each dimension and present the lowest dimension score. This, however, resulted in a clear imbalance towards hyperglycemia and failed to capture the risks that hypoglycemia poses. Therefore, different options were investigated in order to capture a low hypoglycemic score for a minority of days in the period. One alternative was to let the single day with the lowest hypoglycemic score represent the whole time period, that is, in order to give highest priority to the risk of hypoglycemia. However, as expected, this instead tilted the system towards hypoglycemia and lead to an under-representation of hyperglycemia. To balance the two opposing risks, a systematic exploration was conducted in order to investigate the impact of stepwise increasing the number of days to represent the hypoglycemia dimension. Therefore we gradually increased the number of days, in ranked order starting with lowest score, during a 14-day period to represent the hypoglycemia dimension in order to identify the most representative number of days. The hyperglycemic and variability dimensions were both represented by their mean score from all 14 days. The single score was represented by the lowest of the three 14-day dimensional scores.

### Impact of missing data on score

To investigate how representative the scoring is when parts of the data are missing, the data set was filtered to have at least 90% of data per day and at least 90% of data for a period of 30 consecutive days, resulting in 489 sets of 30 consecutive days. This was considered as full data availability and the scoring derived from this ‘full dataset’ was used as a baseline, that is, a ‘true score’. From this data set, two approaches to reduce the data availability were tested. In the first approach, 10%–80% (in steps of 10) of the data were randomly sampled from the full data set, and the score was calculated for each of the ratios and compared with the ‘true score’. In the second approach, full days, in the range of 1 to 29 with a step of 3, of the 30-day period were omitted and the corresponding scores were compared with the ‘true score’. In addition, the consistency of the dimension with the lowest score for each period was investigated in both approaches.

### Score value and CGM treatment targets

In order to determine the scoring value that would represent the fulfillment of all glycemic targets for CGM data according to consensus statements (online supplemental table 1) a receiver operating characteristics (ROC) curve was used combined with an acceptable false positive rate (FPR) of 2.5%. To illustrate the determined limit for reached treatment targets in terms of AGP metrics, linear regression was used between the relevant dimensions of the score and the corresponding AGP metrics. The modeling was conducted as second-degree polynomial models. All 14-day periods with at least 70% data availability (resulting in n=5182 14 -day periods) were used for the ROC curve and modeling.

### Clinical expert board

A CEB consisting of five clinical doctors (two specialists in Pediatrics (HH and ML) and three specialists in Endocrinology and Diabetology (P-OC, JC-C, SBC)) with vast experience in diabetes management and CGM interpretation was assigned five different validation tests of CGM data. All tests were completed individually and the only instructions the CEB received were the design of the test and to evaluate the CGM data based on their clinical judgment. In test 1, the CEB was presented with two daily CGM curves and asked to select which of the two they found most alarming. The pairs of daily glucose curves were selected to have a difference in the respective score of 10–90 (in steps of 10), and represent comparisons within and between each quartile of score. This was repeated in separate set-ups for each of the three dimensions with 32 pairs of CGM curves in each dimension, that is, 96 pairs in total. In test 2, the CEB was presented with 30 daily CGM curves and asked to rate which of the three dimensions (ie, hypoglycemia, hyperglycemia or variability) of glycemic control they found most alarming for each daily curve. Ten daily CGM curves were randomly selected from each of the three dimensions with regard to which dimension had the lowest score for that day. Days with a score margin <10 between lowest dimension score and the second lowest dimension score were excluded. In test 3, the CEB was presented with 30 sets of 14 daily glucose curves combined with an AGP curve and tabular AGP metrics and were asked to rate which of the three dimensions they found most alarming overall. Ten sets of 14-day CGM curves were randomly selected from each of the three dimensions with regard to which dimension had the lowest score for that day. Days with a margin <10 between the lowest dimension score and the second lowest dimension score were excluded. In test 4, the CEB was presented with 50 pairs of 14 daily glucose curves combined with an AGP curve and tabular AGP metrics and were asked to rate which of the two time periods they found most alarming overall. Separately, after a minimum of 4 weeks since the completion of test 4, the CEB was presented with the same test again but without the AGP curve and tabular AGP metrics. However, the evaluators were not informed that test 5 contained the same pairwise comparisons as in test 4. The pairs of 14-day sets were randomly selected, with the condition that the margin between the scores of the sets was at least 10. Apart from the CGM data and AGP metrics, the only additional information they were given was whether the data were derived from an adult or child. All tests were completed online. The inter-rater agreement between evaluators was computed. Based on the individual interpretations, a majority decision was computed (ie, ≥3 ‘votes’) for all questions and compared with that of the scoring algorithm. Examples of questions from each of the tests are presented as supplementary data (online supplemental file 1).

### Statistical analysis

Correlations between scoring values and CGM-derived metrics were computed with Spearman’s rank order test. Values are given as means±SD. Values of p<0.05 were considered statistically significant. The inter-rater agreement within the CEB was calculated as the average of the agreement per question, that is, if all five experts agreed on a question it would correspond to an inter-rater agreement of 100%. For each expert in the board, the agreement with the majority vote was also calculated. The Fleiss’ kappa reliability measure was computed for each of the tests that the CEB completed. For tests 1, 4, and 5 accuracy was defined as the number of questions where the majority vote of the expert panel matched the scoring, divided with the total number of questions. The accuracy for tests 2 and 3 was calculated per dimension (hypoglycemia, hyperglycemia, and variability) using true positive (TP), true negative (TN), false positive (FP), and false negative (FN) according to the formula: Accuracy = (TP+TN) / (TP+TN + FP + FN).

## Results

### Three-dimensional score and a single representative score

**Figure 1:**
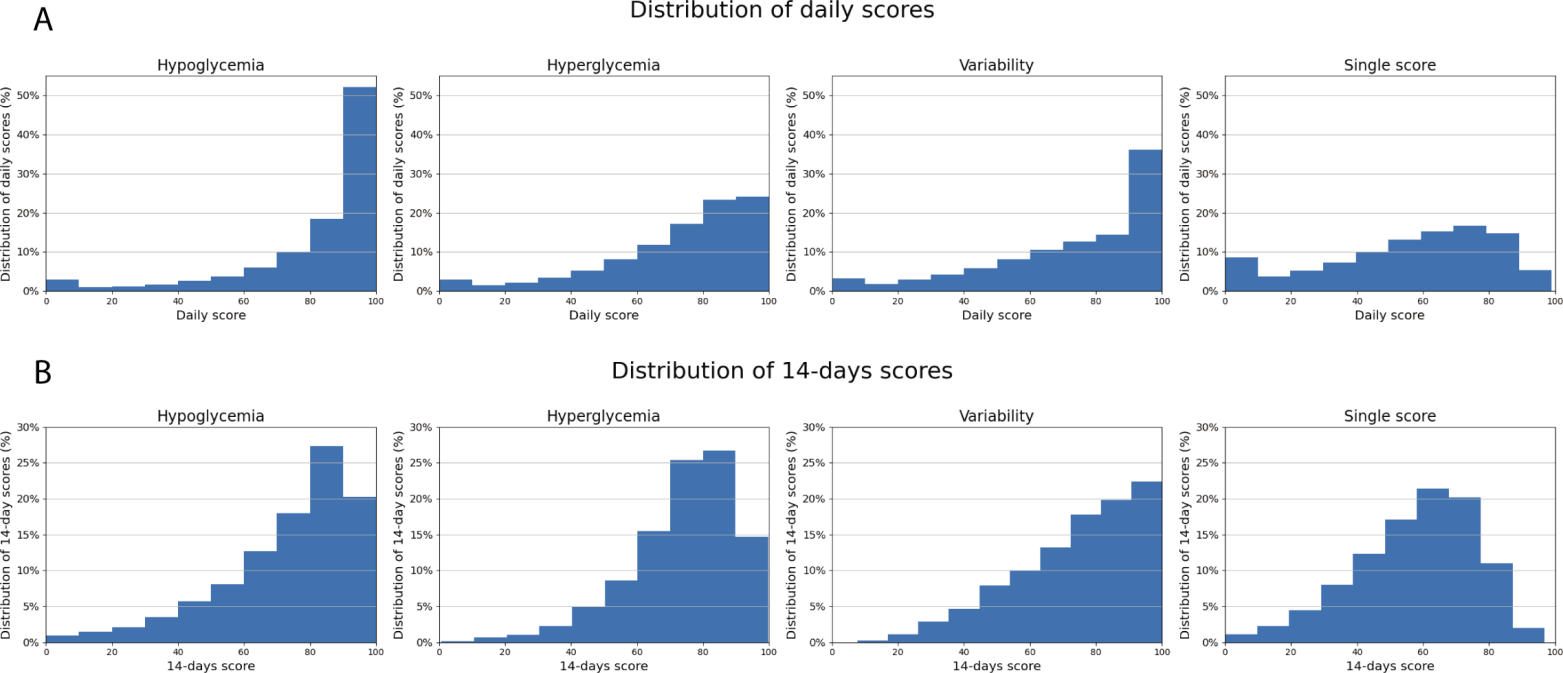
Distribution of the daily (**A**) and aggregated 14-day score (**B**) for each of the three dimensions, that is, hypoglycemia, hyperglycemia, and variability, from the full study cohort and the computed single score computed.

The resulting distributions of the three dimensions of scores are presented in figure 1.

**Figure 2:**
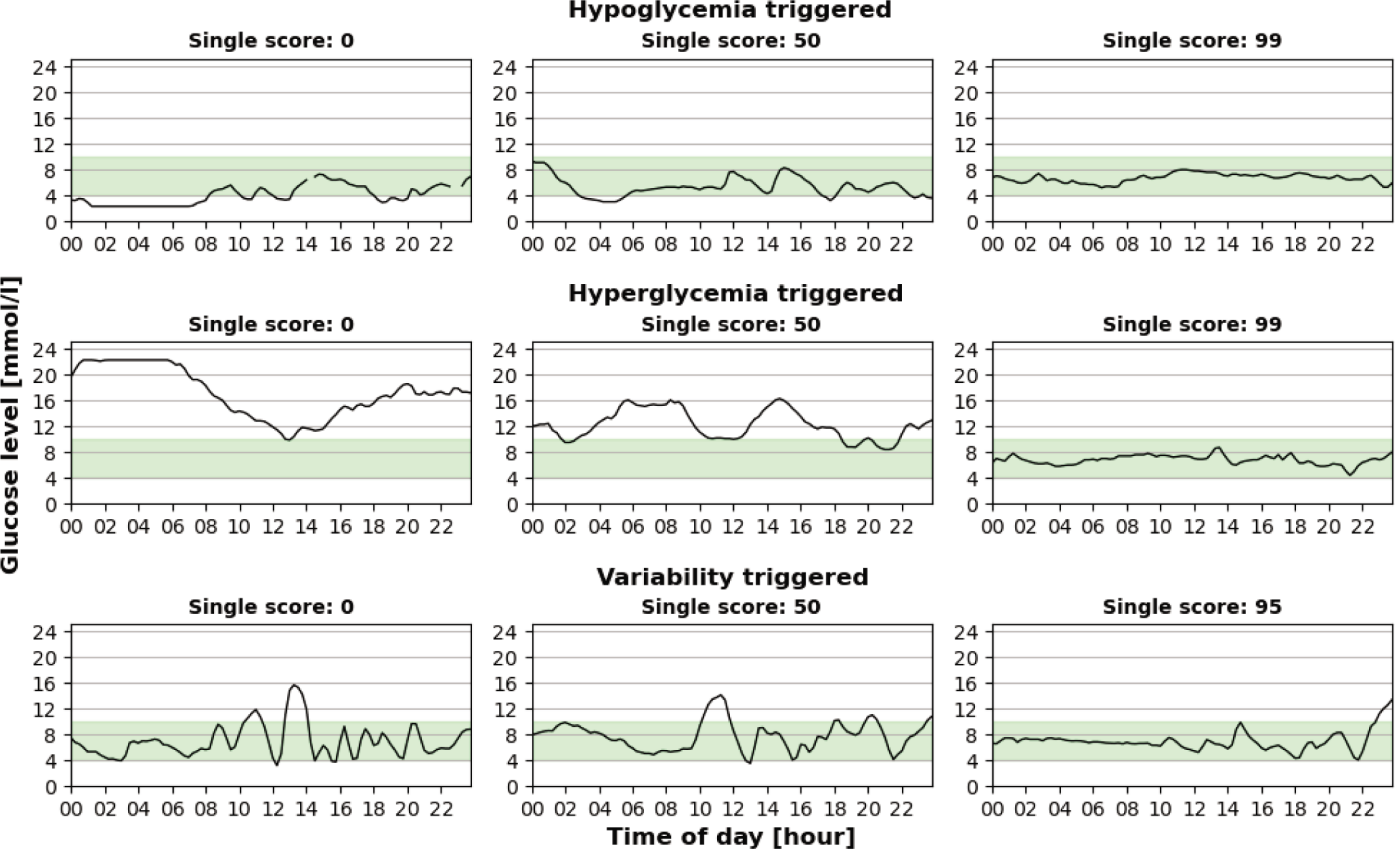
Example of daily glucose curves with a gradually increasing score for each of the three dimensions.

**Figure 3:**
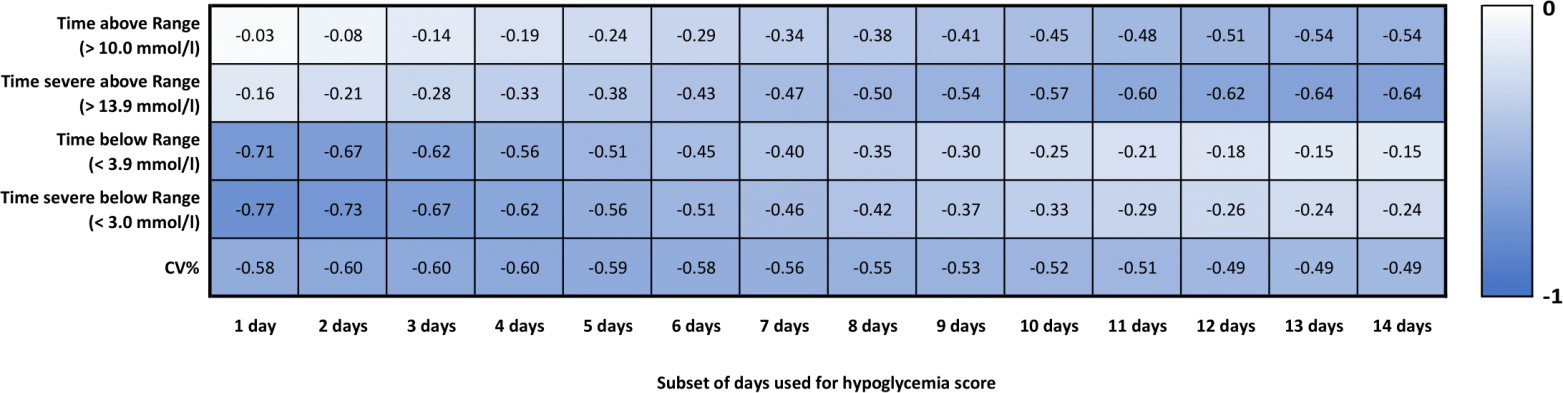
Correlations between ambulatory glucose profile (AGP) metrics and the single score for a 14-day time period with increasing number of days to represent the hypoglycemia dimension. CV%, coefficient of variation.

Since the data were normalized so that a score of 0 would represent the most alarming 2% (*ie* > 98th percentile) of the cohort for that dimension, it is to be expected that daily CGM curves with a low score for a certain dimension would be far from optimal. To visualize this, five daily curves were plotted for each dimension with scoring values of 0, 25, 50, 75, and 99 in figure 2. For each of the curves, this was the dimension with the lowest score. However, since all three dimensions were computed, it is still possible that the same daily curve would have a low (but not lower) score for more than one dimension. When gradually increasing the number of days to represent the mean for the hypoglycemia dimension we found that the correlation of both TAR and TBR was balanced (r=-0.38 *vs* −0.35) when 8 days were selected. However, a 7-day representation resulted in a better balance between TSAR and TSBR (r=-0.47 *vs* −0.46). Based on that, a subset size of 50% of the lowest hypoglycemic scoring days was used in the final scoring model, that is, for computing the hypoglycemia score for extended time periods. The single score for each stepwise addition of days to represent the hypoglycemic dimension was correlated with TAR, TSAR, TBR, TSBR, and CV% and is presented in figure 3.

### Correlation between score and AGP metrics

**Figure 4:**
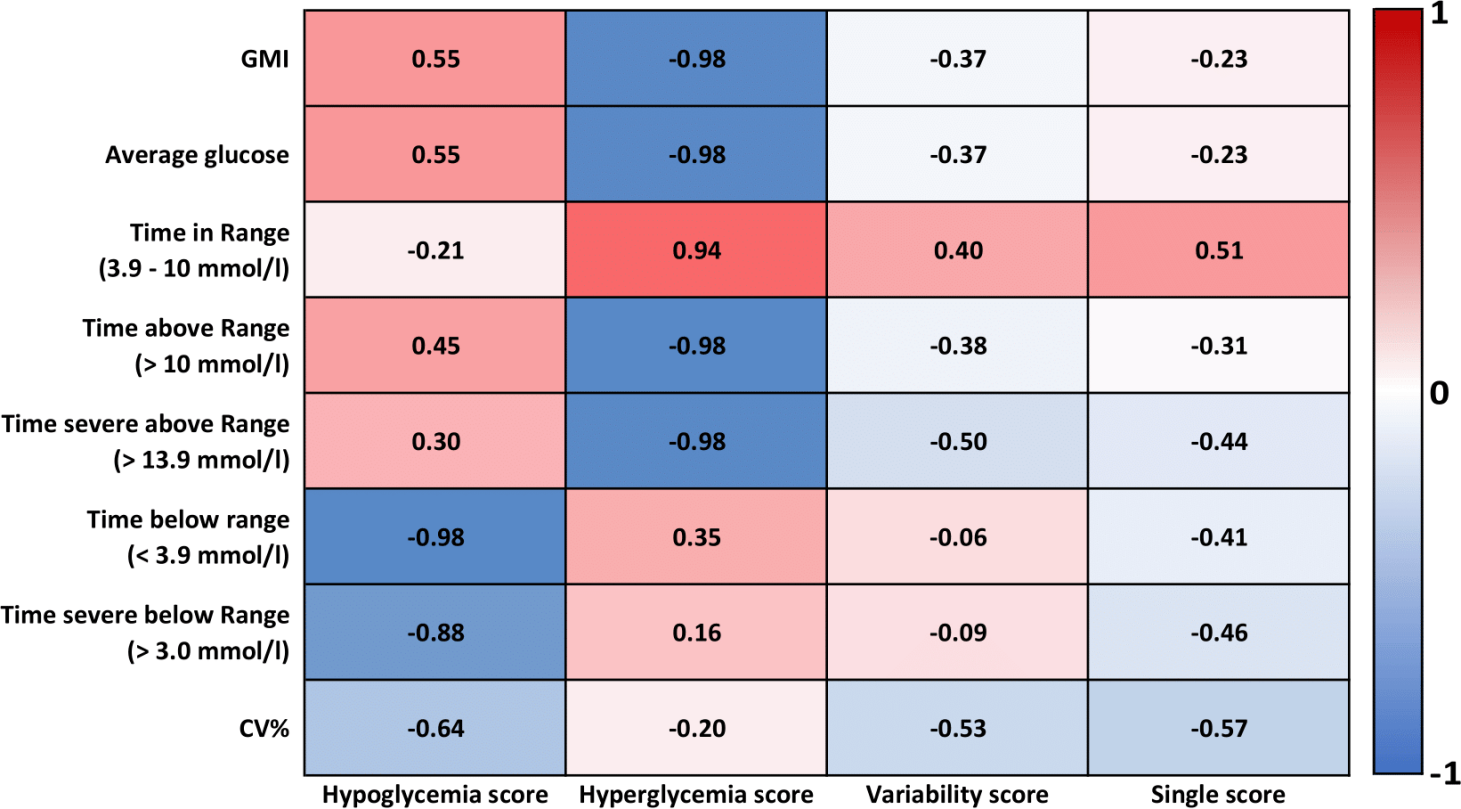
Correlation matrix of ambulatory glucose profile (AGP) metrics and the three dimensions of score, that is, hypoglycemia, hyperglycemia, variability and the single score computed from n=5182 time series of 14 days derived from n=613 subjects with type 1 diabetes (T1D). CV%, coefficient of variation.

The correlations between score and commonly used AGP metrics were computed from n=5182 14-day series derived from the n=613 subjects with T1D. All the dimension scores correlated with the listed AGP metrics (p<0.0001). As expected, the hypoglycemia and hyperglycemia scores correlated with the corresponding AGP metrics (ie, time below range (r=−0.98) and time above range (r=−0.98) respectively). The hyperglycemia score also correlated strongly with Glucose Management Indicator (GMI) (r=−0.98) and average glucose (r=−0.98). The variability score correlated with CV% (r=−0.53) but due to the differences of GVP and CV% the value of r was low. The variability score also had a weak, but statistically significant, correlation with TBR (r=−0.06) and TSBR (r=−0.09). The single score from 14 days of data correlated with all listed AGP metrics (p<0.0001) but was evenly correlated with TIR (r=0.52), TSAR (r=−0.44), TSBR (r=−0.46), and CV% (r=−0.57). All correlations are presented in figure 4.

### Impact of missing data on score

The differences in score for periods of 30 days of CGM data with a simulation of increasingly random missing data compared with the full data sets are presented in online supplemental figure 1. A positive difference in the histogram represents a falsely higher score. The impact of randomly reducing the data availability was surprisingly modest even down to a 50% reduction. In general, the reduction of data resulted in a falsely improved score. The accuracy for the dimension of glucose control that triggered the lowest score was surprisingly stable with a scoring dimension accuracy of 73% even when only 50% of data were available (online supplemental figure 1). Similarly, when randomly decreasing full days of data the impact on score was modest. Even when only 14 days out of 30 days were available the scoring dimension accuracy was still 83%.

### Scoring value corresponding to treatment targets

**Figure 5:**
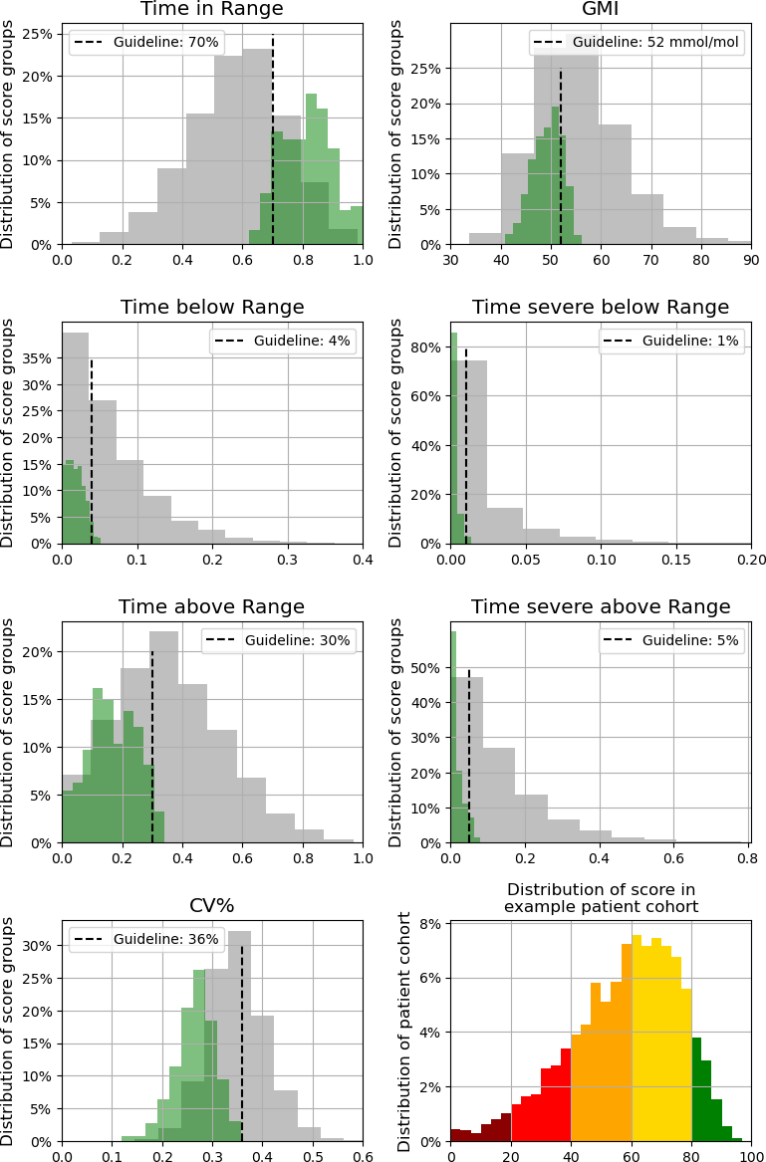
Identification of cut-off values for the scoring model. (**A**) Receiver operating characteristics (ROC) curve was applied in order to identify the numeric scoring value which would correspond to a high likelihood of fulfilling all target values for continuous glucose monitoring (CGM) data. Based on the data the cut-off limit was set to a scoring value of ≥80 which is visualized by the color green. This resulted in an accuracy of 93.4% when compared with a ‘true’ target fulfillment as previously described. The specificity was 97.1%, which means that very few CGM periods would be falsely classified as green. The sensitivity was 61.4%, showing that the score is a stricter measure of glucose control than commonly used AGP metrics. (**B**) Distributions of commonly used AGP metrics and score. The green segment represents the highest scoring segment in which the likelihood of fulfilling the treatment targets is high. The dotted lines indicate the currently applied target value for each of the AGP metrics. Patients with a score <80 are represented by gray for visualization purposes in order to highlight the relationship to target values in the green segment. The graph in the lower right corner displays the distribution of score for the whole cohort grouped into five color-coded (in intervals of 20) segments. Dark red and red represent the two lowest segments of score and are hence furthest from achieving the treatment guidelines for CGM data. The majority of the patients are found in the orange and yellow segments (ie, with a score between 40 and 79). AGP, ambulatory glucose profile; AUC, area under the curve; CV%, coefficient of variation; FPR, false positive rate.

The ROC curve of the score compared with the fulfillment of all consensus guidelines for CGM metrics (outlined in online supplemental table 1) displayed an area under the curve of 0.91 (figure 5A). An FPR of 2.5% corresponded to a score of 80.3 and a true positive rate of 60.7%. Applying a score of ≥80 as the cut-off resulted in an accuracy of 93.4% when compared with a ‘true’ target fulfillment as previously described. The specificity was 97.1%, which means that very few CGM periods would be falsely classified as fulfilling the treatment targets. The sensitivity was 61.4%, showing that the score is a stricter measure of glucose control than commonly used AGP metrics. Based on that, a score of ≥80 was assigned to represent the likely fulfillment of all treatment targets, visualized in the histogram by the color green (figure 5B). The remaining interval of score (ie, 0–79) was divided into four groups, that is, in segments of 20. The lowest score segment (0–19) was assigned a dark red color and the following segment (20–39), which is still far from treatment targets, was also assigned a red color for visualization purposes. The score segment of 40–59 was assigned an orange color and the segment of 60–79 a yellow color. The distribution of the single score in relation to each of the relevant AGP metrics, divided into the green and ‘non-green’ segments, are visualized in figure 5B. When plotting the distribution of all score segments, visualized by their respective color, it becomes apparent that most patients fall into the yellow and orange segments (ie, score 40–79) (figure 5B).

All scores based on 14-day time periods in the patient cohort and the corresponding linear regression polynomial models for TBR, TSBR, TAR, TSAR, TIR, CV%, and GMI are presented in online supplemental figure 2. All models, except for the variability score and CV%, resulted in a good fit with an r^2^ higher than 0.85. The lower r^2^ (0.29) for the variability score and CV% was expected due to the inherent differences of CV% and GVP. The AGP metrics derived from these linear regressions for each segment of score are presented in online supplemental table 2.

### Clinical expert board

In *test 1*, the CEB performed pairwise comparisons of daily CGM curves and determined which day they found most alarming for each of the three dimensions (ie, hypoglycemia, hyperglycemia, and variability). For this test, the accuracy of the scoring algorithm was 87.5% for hypoglycemia, 96.9% for hyperglycemia, and 75.0% for variability when compared with the CEB majority vote, resulting in an overall accuracy of 86.5% (table 2). The overall value for Fleiss’ kappa reliability within the CEB was 0.61, corresponding to a ‘substantial’ level of agreement. The overall inter-rater agreement in the CEB was 89%. In *test 2*, when evaluating daily CGM curves in order to determine which of the three dimensions the CEB found most alarming, the accuracy of the scoring algorithm was 88.5% for hypoglycemia, 80.8% for hyperglycemia, and 69.2% for variability compared with the majority vote of the CEB (table 2). The overall inter-rater agreement in the CEB was 72%. The value for Fleiss’ kappa reliability was 0.29, corresponding to a ‘fair’ level of agreement. The number of answers that differed from the majority vote for each rater was on average 10 out of 30 and the agreement with the majority for each rater was on average 66.7%. In *test 3*, the CEB evaluated sets with 14 daily CGM curves combined with a summative AGP curve including tabular AGP metrics and were asked to determine which dimension they found most alarming overall. For this test, the accuracy of the scoring algorithm was 74.1% for hypoglycemia, 92.6% for hyperglycemia, and 66.7% for variability when compared with the majority vote of the CEB (table 2). The value for Fleiss’ kappa reliability was 0.38 which corresponds to a ‘fair’ level of agreement. In *test 4*, which consisted of a pairwise comparison between two sets of 14 daily CGM curves combined with a summative AGP curve and tabular AGP metrics, the overall accuracy of the scoring algorithm was 68.0% when compared with the majority vote of the CEB. The inter-rater agreement in the CEB was high (93.2%) and the value for Fleiss’ kappa reliability was 0.76, corresponding to a ‘substantial’ level of agreement. When conducting the same test again but without access to AGP metrics (*test 5*), the accuracy between the scoring algorithm and CEB was 74%. The inter-rater agreement within the CEB was lower (88.4%), resulting in a lower Fleiss’ kappa reliability (0.61) but still corresponding to a ‘substantial’ level of agreement.

**Table 2:** Accuracy of the scoring algorithm when compared with the majority vote of the clinical expert board

Parameter	Scoring accuracy (%)
Test 1	
Hypoglycemia	87.5
Hyperglycemia	96.9
Variability	75.0
Overall	86.5
Test 2	
Hypoglycemia	88.5
Hyperglycemia	80.8
Variability	69.2
Test 3	
Hypoglycemia	74.1
Hyperglycemia	92.6
Variability	66.7
Test 4	
Overall	68.0
Test 5	
Overall	74.0

## Discussion

There have been several attempts to develop a composite score for CGM data, either to reflect overall glucose control or the risk for hypoglycemia or hyperglycemia.^16^ In general, metrics are computed either based on raw data, that is, the time series of individual glucose values, or based on AGP metrics. Since the AGP report has gained such a high appreciation in the clinical setting and has been adopted by consensus guidelines, these metrics are in most studies used as a reference for newly developed metrics. A common problem with metrics intended to capture the complexity of overall glucose control is that they correlate well with TIR or GMI but fail to capture the risk of hypoglycemia. On the other hand, metrics developed to capture the risk of hypoglycemia often fail to differentiate between patients with a low risk of hypoglycemia but varying degrees of hyperglycemia.

In a recent study by Klonoff *et al* a different approach was used for developing a Glycemia Risk Index (GRI) of both hypoglycemia and hyperglycemia.^17^ Based on 14-day CGM tracings from n=225 patients they developed a separate hypoglycemia and hyperglycemia component and a composite score. The algorithm weighs glucose abnormalities in order to penalize very low and very high values based on a five-step function. This is similar to the LBGI/HBGI, which however has a smoothing function that prevents small alterations of glucose to have dramatic effects on the index. Although the GRI correlates well with the standardized AGP metrics, it was not designed to capture variations of glucose but rather to reflect CGM readings in which severe hypoglycemia or severe hyperglycemia episodes are already present. Also, there is increasing evidence suggesting that 14-day readings of CGM data may not be sufficient in order to capture the risk of hypoglycemia and glucose variability.^18^

In the development of our scoring algorithm, we used a large cohort of patients with CGM data, including both adults and children, which extended far beyond 14 days for each patient. Our scoring model combines both LBGI and HBGI in order to capture the risk of hypoglycemia and hyperglycemia and in addition, GVP, in order to capture the glucose variability. In contrast to CV%, the currently most widely adopted metric for glucose variability, GVP, captures not only the amplitude of glucose variations but also the frequency. GVP can simply be explained as a function depending on the ‘length’ of the CGM curve which will therefore increase even if the glucose fluctuations are limited to within the TIR (3.9–10 mmol/L).^10^ This makes it possible to capture both, patients that already have either alarming features of hypoglycemia or hyperglycemia, and those with a high variability, which both can increase the risk of severe hypoglycemia and negatively impact their daily life. By normalizing the data and weighing the hypoglycemia feature, we can also present the single score that most adequately reflects the most alarming dimension of glucose control for each patient (or different time periods for the same patient). Hence, if a high single score is achieved, this would still reflect the ‘most alarming’ dimension for that patient, meaning that the other aspects of glucose control are even ‘better’. This reduces the risk of falsely assigning a high score to a patient while also ensuring that those who have a high score in fact most likely fulfill all treatment targets for glycemia. By applying this method, we reduce the risk of falsely labeling a patient as having a high score simply due to the absence of severe hypoglycemia and/or hyperglycemia.

As expected, the dimensions of hypoglycemia and hyperglycemia correlated well with the respective AGP metrics of relevance. However, the variability score did not correlate as well to CV% or any of the other AGP metrics. This can be explained by the inherited differences of GVP and CV%, since GVP accounts for both the amplitude and frequency of glucose excursions whereas CV% is only affected by the amplitude of excursions. This also translates into the identification of a cut-off value for the scoring model that would reflect a high likelihood of fulfilling all the CGM targets for which the CV% in the ‘green’ group is ≤34%, that is, lower than the proposed target of ≤36% as in current treatment guidelines. However, recent publications have suggested that a stricter criterion for CV% could decrease the risk of severe hypoglycemia, notably, the proposed level was in fact 34%.^19^ When the CEB evaluated CGM curves we found that the accuracy for identifying the most alarming daily curve within each dimension was highest for hyperglycemia (97%) and lowest for variability (75%) which could reflect that clinicians are trained to think of variability as a result of the amplitude of glucose excursions. The same phenomenon was observed in the other tests completed by the CEB. When the CEB reviewed two different 14-day periods of CGM data and AGP metrics (ie, test 4) the inter-rater agreement was surprisingly high (93%) which likely reflects a great emphasis on the tabular AGP metrics. Interestingly, when completing the same test more than 4 weeks later without access to AGP metrics the inter-rater agreement was lower (88%) and the accuracy when compared with the scoring model was higher (74% *vs* 68%). This could reflect a difference in how clinicians interpret daily CGM curves as opposed to aggregated AGP metrics with defined target values. It should also be noted that a large number of the comparisons were rather similar in terms of glycemia, that is, with a low difference in score, which of course also affected the results.

A strength of the current study is the fairly vast amount of available CGM data which includes far beyond the standard 14-day time period for all patients. In addition, the patient cohort includes both pediatric and adult patients with T1D. A limitation on the other hand is that even though patient data were collected from both adult and pediatric patients, they were all from the same university hospital. This could be a limitation in terms of the normalization of data since the distribution may not be representative for other cohorts. However, since the treatment guidelines for diabetes management and CGM data are international it is still highly likely that the adopted cut-off value for a score to represent the fulfillment of all treatment targets is valid since this was related directly to the treatment targets and not the normalization. In addition, the study is based on retrospective data and although the scoring algorithm has been validated against the interpretation of a CEB there is still need for further prospective clinical validation studies.

By adopting the scoring algorithm in clinical practice, it would be possible to continuously track a large cohort of patients based on their CGM data. With this approach, alterations over time for individual patients could easily be detected but this would also make it possible to stratify the need for clinical attention for a whole cohort of patients. In contrast to current practice, at most diabetes clinics a ‘CGM-centric workflow’ could be adopted, that is, to plan the care for patients based on the current glucose status rather than based on a predefined static interval for follow-up. In order to facilitate this, the access to CGM data is crucial but, if available, patients could be stratified based on their current score, ensuring that those in most need of attention are scheduled for more frequent visits, whereas those with a high score can be maintained on the waiting list for their annual visits as planned. By applying this workflow, a clinic could ideally identify the patients in most need of medical attention, and in addition their main glycemic challenge, in order to assist them with improving that aspect early on rather than waiting until their next scheduled visit. The implementation of a scoring algorithm could also make it possible to identify groups of patients with similar challenges, in order to direct targeted interventions. For instance, it could facilitate the implementation of targeted intervention for patients with a low score due to hypoglycemia, to ensure that all available clinical measures have been offered to every patient in that group. Ideally, this could be organized as checklists which would ensure that the available measures are in fact put into place.

## Conclusion

We found that our scoring algorithm performs as well as clinical experts both in terms of identifying the most alarming dimension of glycemia and the time period with the most alarming glycemic profile. A threshold for the normalized single score can be identified that with high certainty reflects the fulfilment of all CGM-based criteria according to current treatment guidelines. In addition, we observed that the scoring algorithm is robust even for time periods with low data availability. In other words, our scoring algorithm can be used to stratify patients in need of medical attention based on CGM data with an accuracy similar to that of a CEB.

## Supplementary material

10.1136/bmjdrc-2024-004350 online supplemental file 1

10.1136/bmjdrc-2024-004350 online supplemental figure 1

10.1136/bmjdrc-2024-004350 online supplemental figure 2

10.1136/bmjdrc-2024-004350 online supplemental table 1

10.1136/bmjdrc-2024-004350 online supplemental table 2

## Data Availability

Data are available upon reasonable request.
