# Supplementary material for: Development of a three-dimensional scoring model for the assessment of continuous glucose monitoring data in type 1 diabetes
Source: BMJ Open Diabetes Res Care. 2024 Sep 5;12(4):e004350. doi: 10.1136/bmjdrc-2024-004350 (PMC11381645; doi:10.1136/bmjdrc-2024-004350)
Supplement: online supplemental file 1 [file bmjdrc-12-4-s001.pdf]

Test 1. Daily CGM curve rankings

1A.16 *

Which of these days is most alarming regarding **hypoglycemia**?

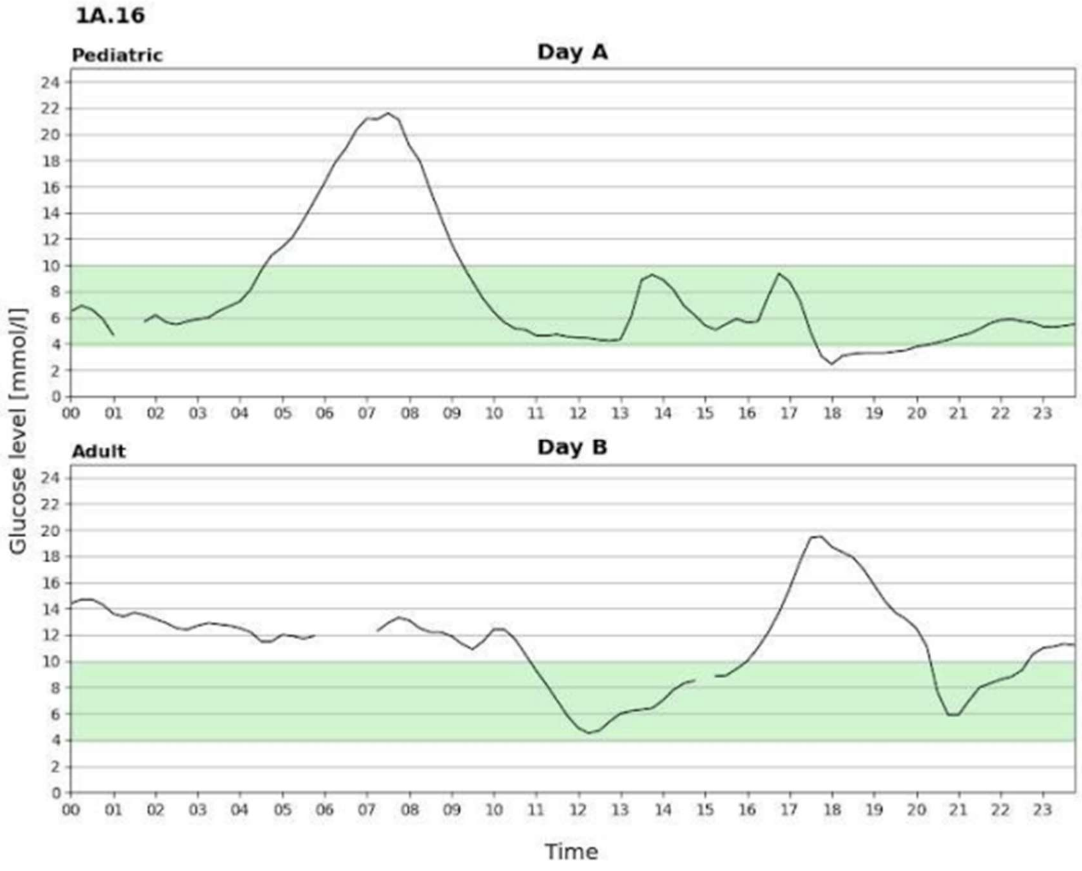

- ○ Day A
- ○ Day B

Day A: Hypoglycemic score of 69

Day B: Hypoglycemic score of 99

1B.16 *

Which of these days is most alarming regarding **hyperglycemia**?

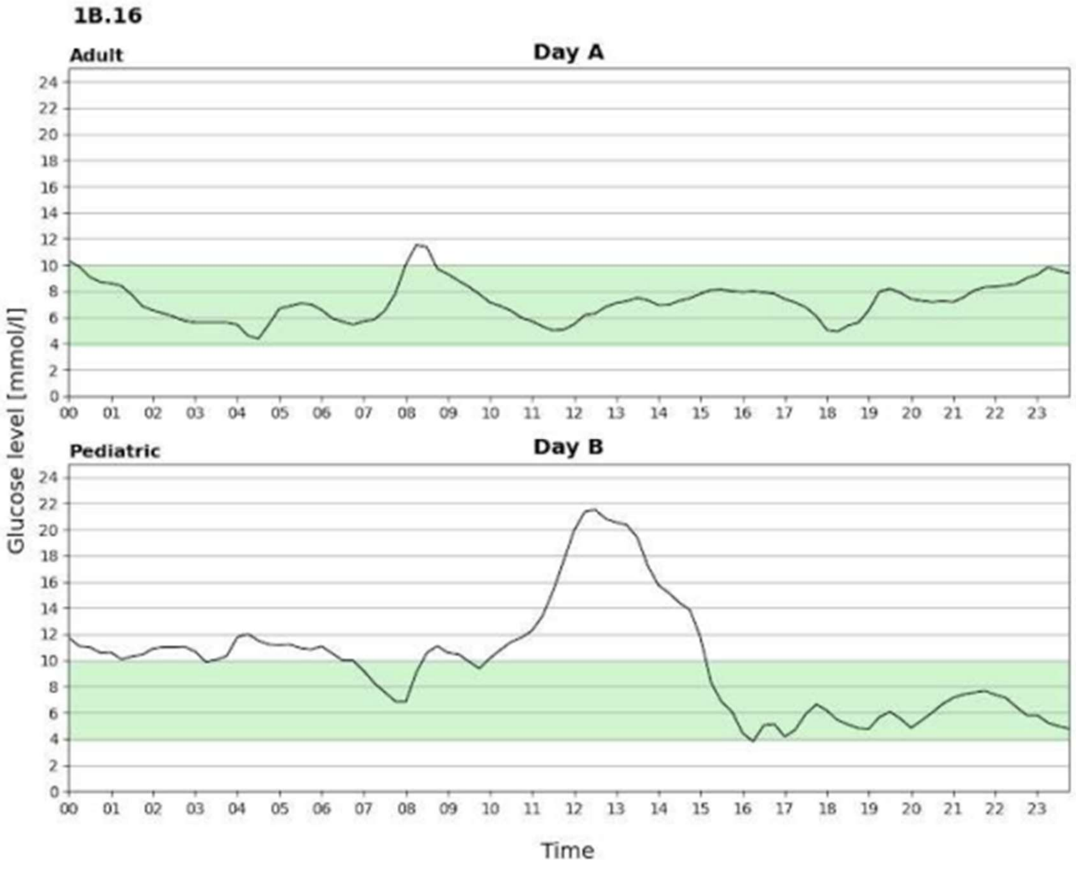

○ Day A

○ Day B

Day A: Hyperglycemic score of 94

Day B: Hyperglycemic score of 64

1C.16 *

Which of these days is most alarming regarding **variability**?

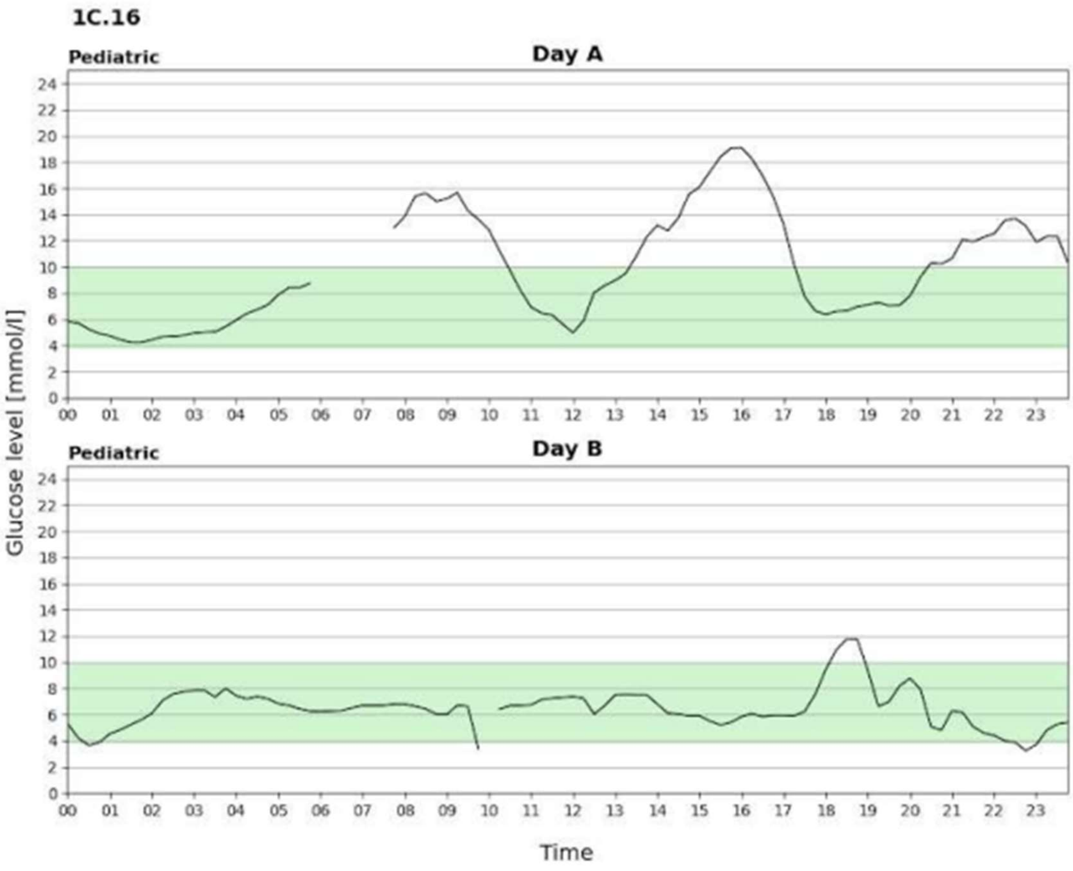

◯ Day A

◯ Day B

Day A: Variability score of 55

Day B: Variability score of 85

Test 2. Most alarming aspect of daily CGM curves

2.1 *

Which of the aspects
- hypoglycemia
- hyperglycemia
- variability

is the most alarming for this day?

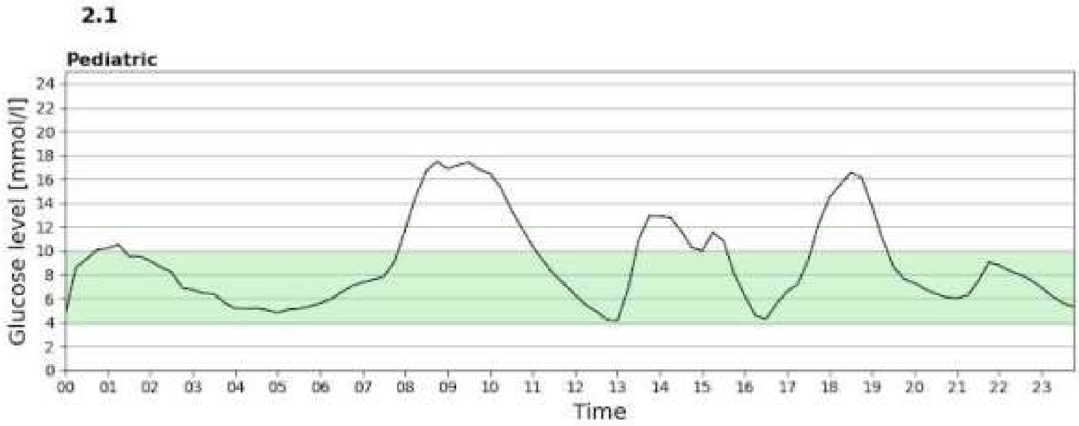

- ○ Hypoglycemia
- ○ Hyperglycemia
- ○ Variability

Hypoglycemic score: 95

Hyperglycemic score: 74

Variability score: 8

2.2 *

Which of the aspects

- hypoglycemia
- hyperglycemia
- variability

is the most alarming for this day?

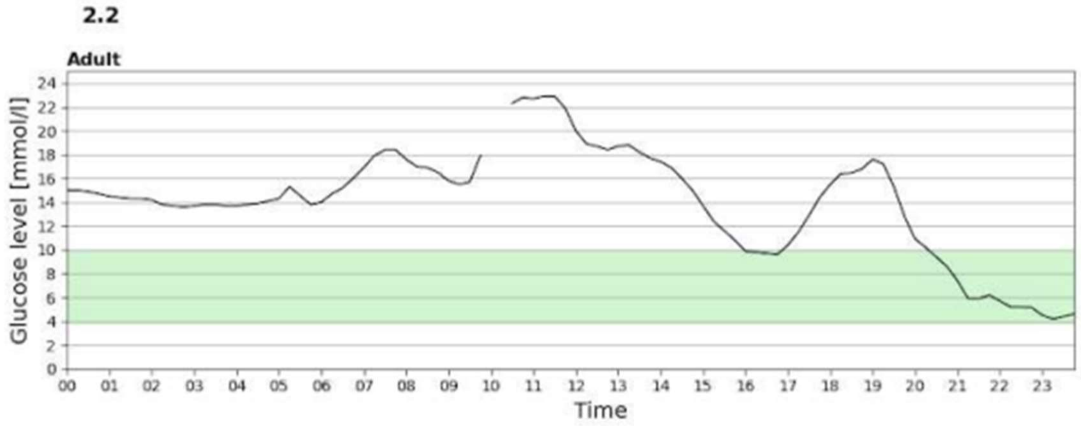

○ Hypoglycemia

○ Hyperglycemia

○ Variability

Hypoglycemic score: 97

Hyperglycemic score: 21

Variability score: 78

2.3 *

Which of the aspects

- hypoglycemia
- hyperglycemia
- variability

is the most alarming for this day?

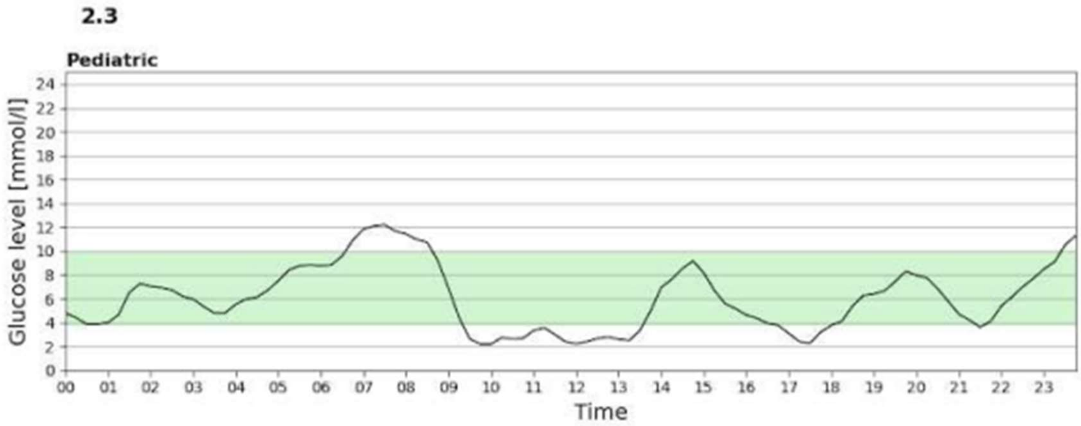

○ Hypoglycemia

○ Hyperglycemia

○ Variability

Hypoglycemic score: 17

Hyperglycemic score: 93

Variability score: 72

Test 3. Most alarming aspect of 14-days CGM curves

3.1 *

Which of the aspects

- hypoglycemia
- hyperglycemia
- variability

is the most alarming for this 14-days period?

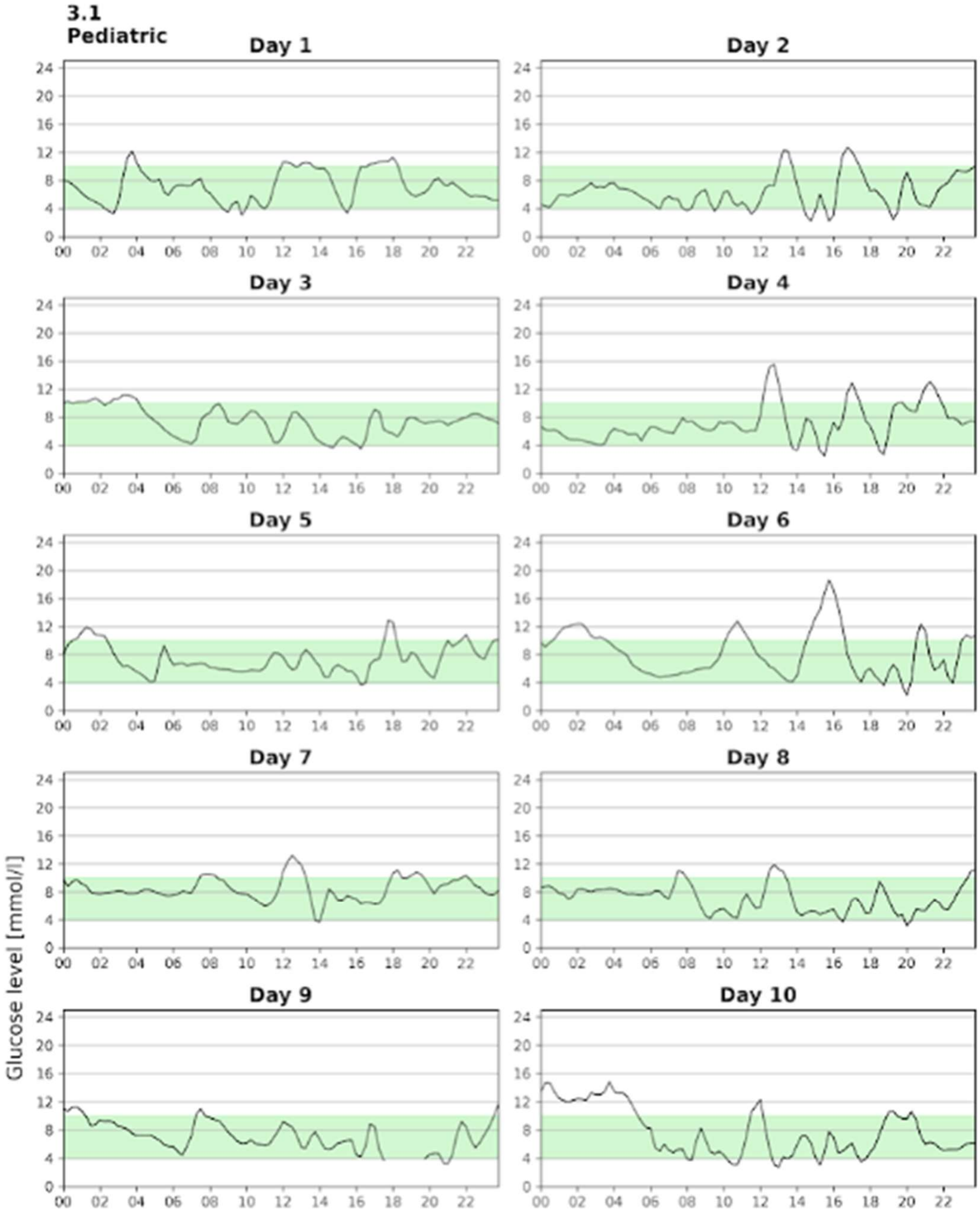

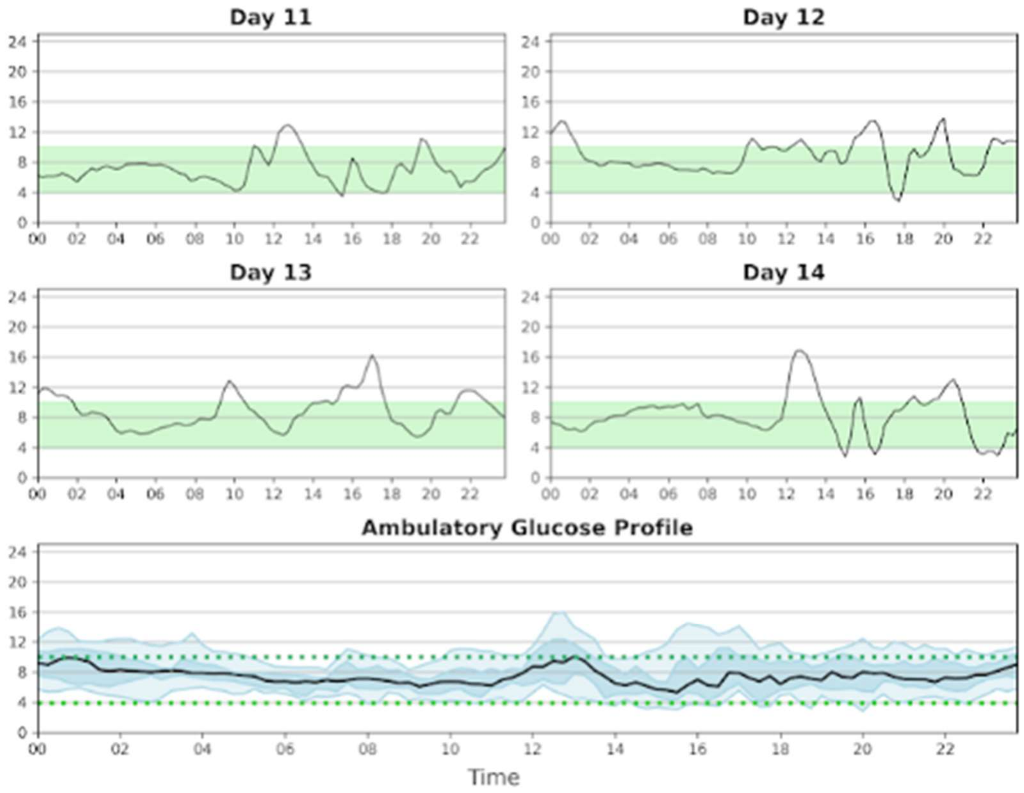

> 13.9 mmol/l:	1%	GMI: 49 mmol/l	
10.0-13.9 mmol/l:	17%	Avg glucose: 7.7 mmol/l	
3.9-10.0 mmol/l:	78%	CV: 33.1%	
3.0-3.9 mmol/l:	4%		
< 3.0 mmol/l:	1%		

○ Hypoglycemia

○ Hyperglycemia

○ Variability

Hypoglycemic score: 78

Hyperglycemic score: 88

Variability score: 37

3.2 *

Which of the aspects

- hypoglycemia
- hyperglycemia
- variability

is the most alarming for this 14-days period?

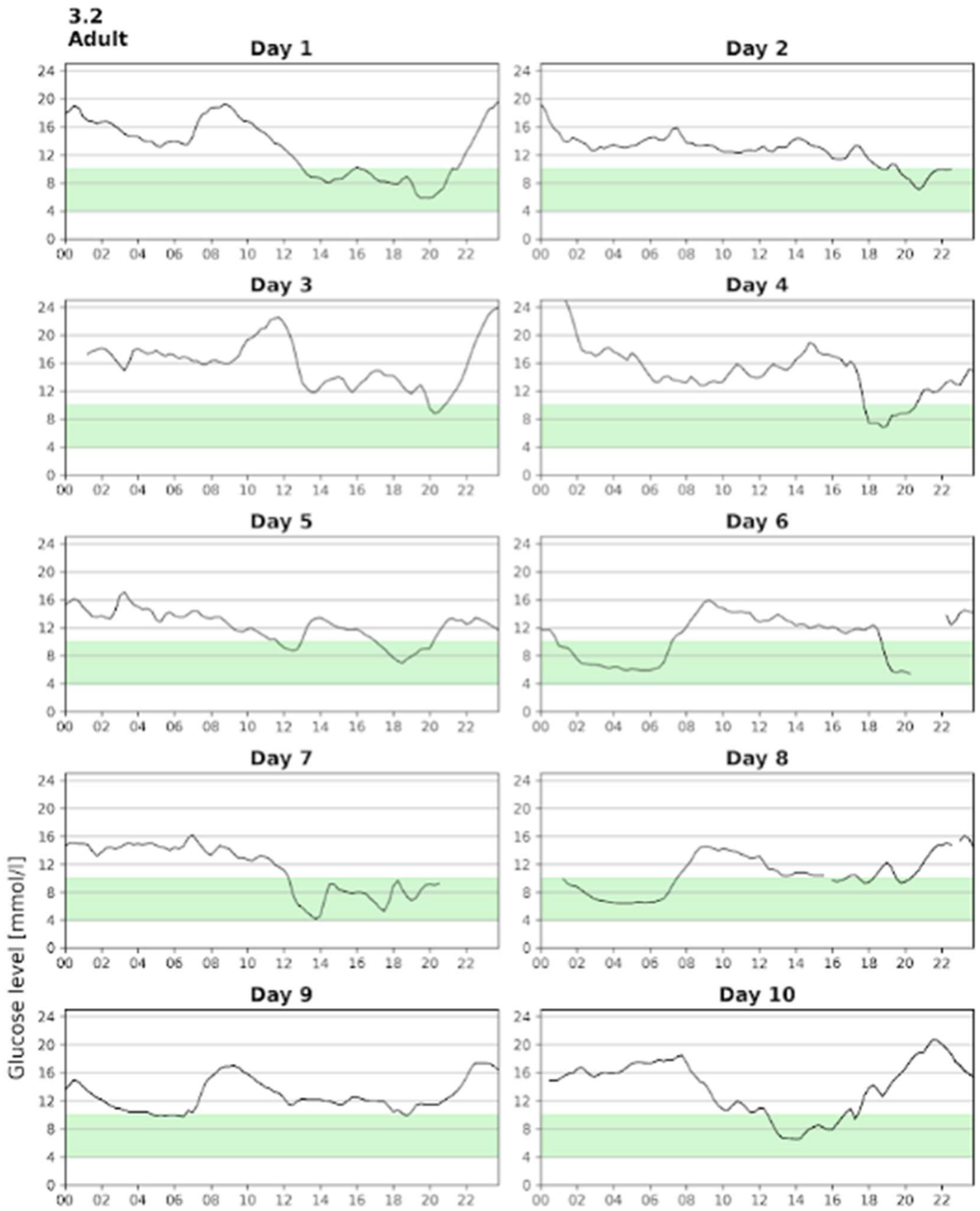

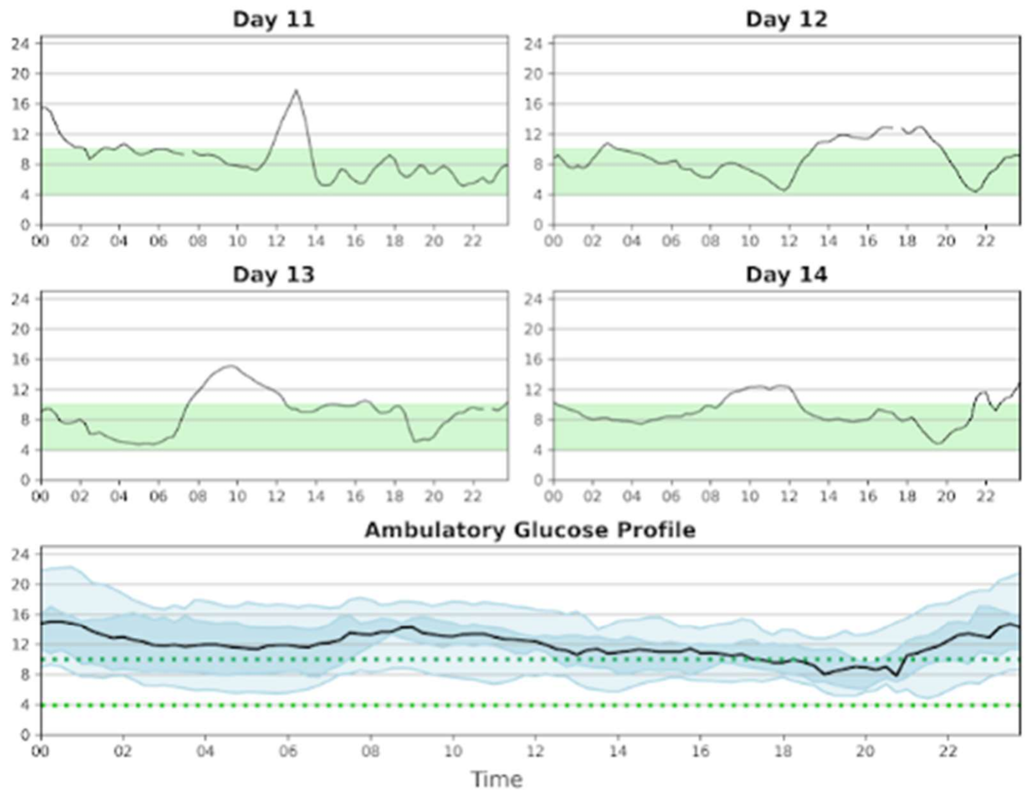

> 13.9 mmol/l:	28%	GMI: 68 mmol/l	
10.0-13.9 mmol/l:	35%	Avg glucose: 11.7 mmol/l	
3.9-10.0 mmol/l:	37%	CV: 32.3%	
3.0-3.9 mmol/l:	0%		
< 3.0 mmol/l:	0%		

- ◯ Hypoglycemia
- ◯ Hyperglycemia
- ◯ Variability

Hypoglycemic score: 98

Hyperglycemic score: 51

Variability score: 90

3.3 *

Which of the aspects

- hypoglycemia
- hyperglycemia
- variability

is the most alarming for this 14-days period?

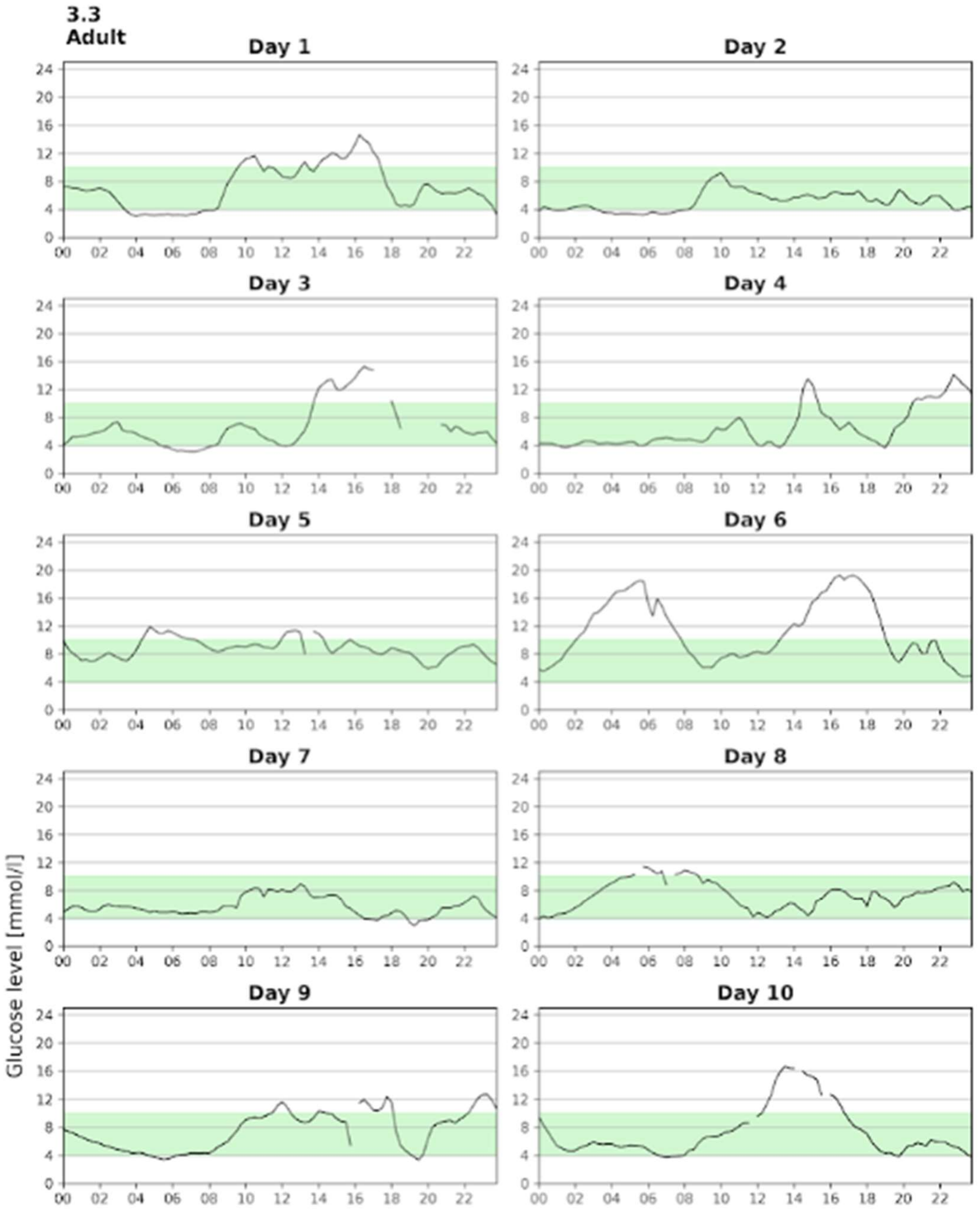

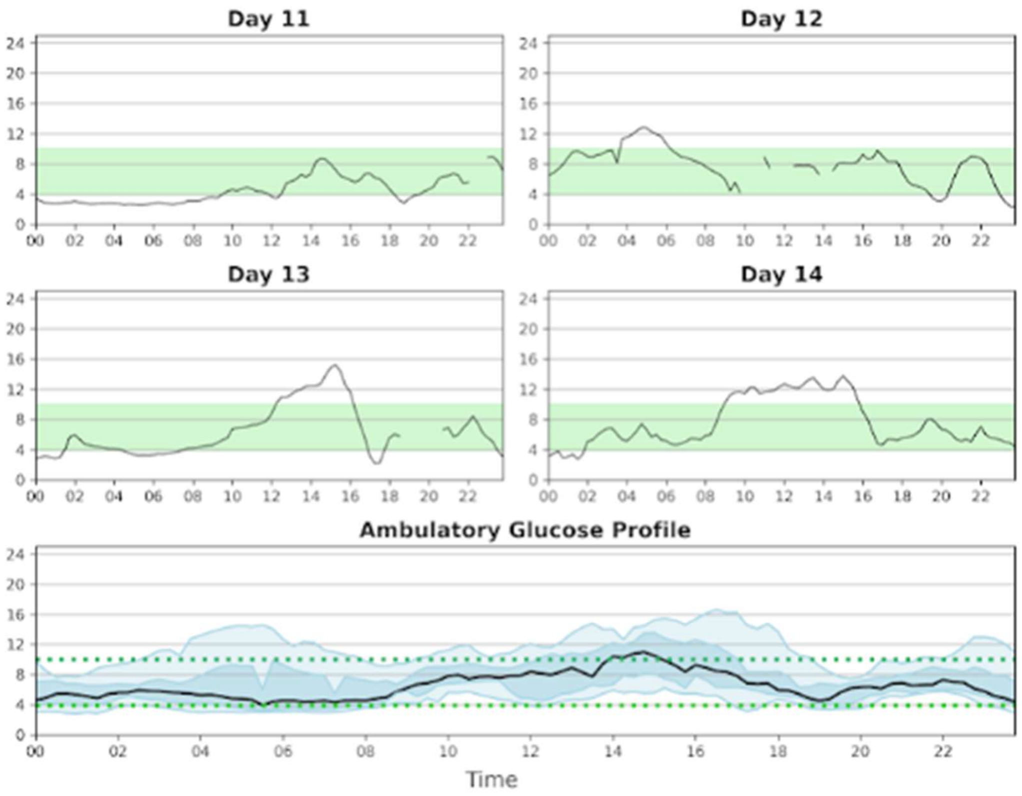

**> 13.9 mmol/l:**	**4%**
**10.0-13.9 mmol/l:**	**14%**
**3.9-10.0 mmol/l:**	**70%**
**3.0-3.9 mmol/l:**	**10%**
**< 3.0 mmol/l:**	**3%**

GMI: 46 mmol/l
Avg glucose: 7.1 mmol/l
CV: 44.9%

- ○ Hypoglycemia
- ○ Hyperglycemia
- ○ Variability

Hypoglycemic score: 49

Hyperglycemic score: 88

Variability score: 89

Test 4. 14-days CGM curves ranking

4.1 *

Which of these two 14-days periods is most alarming?

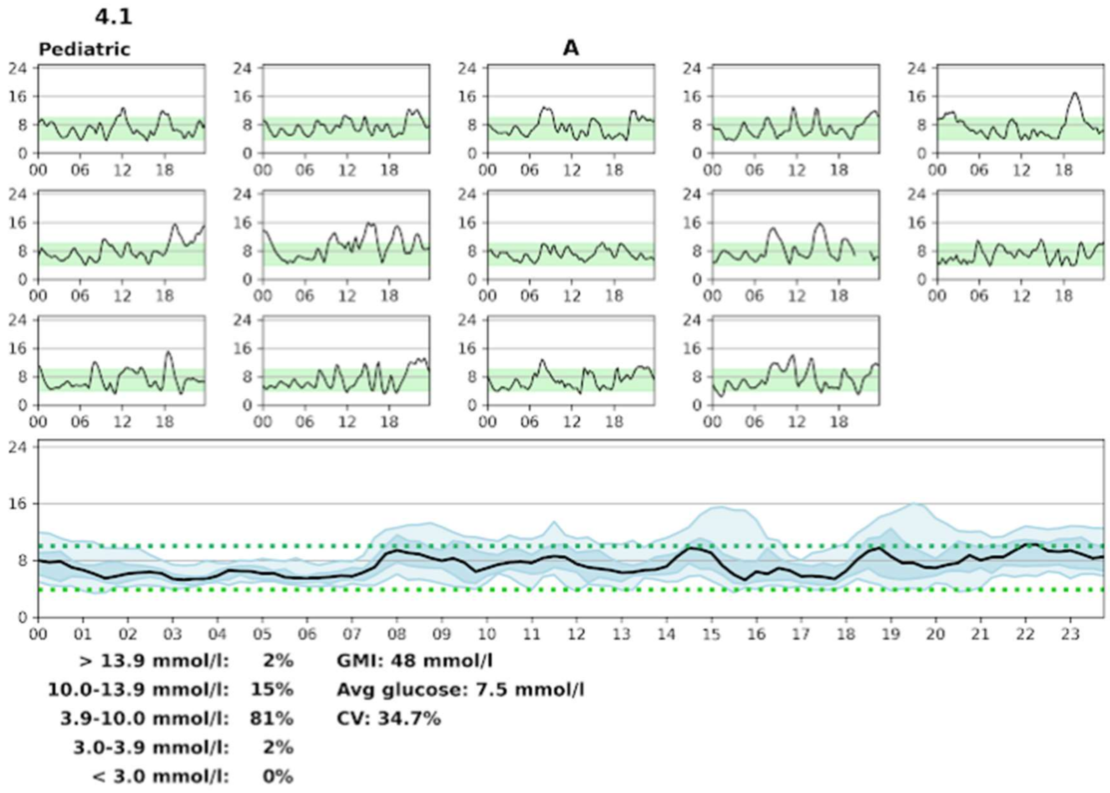

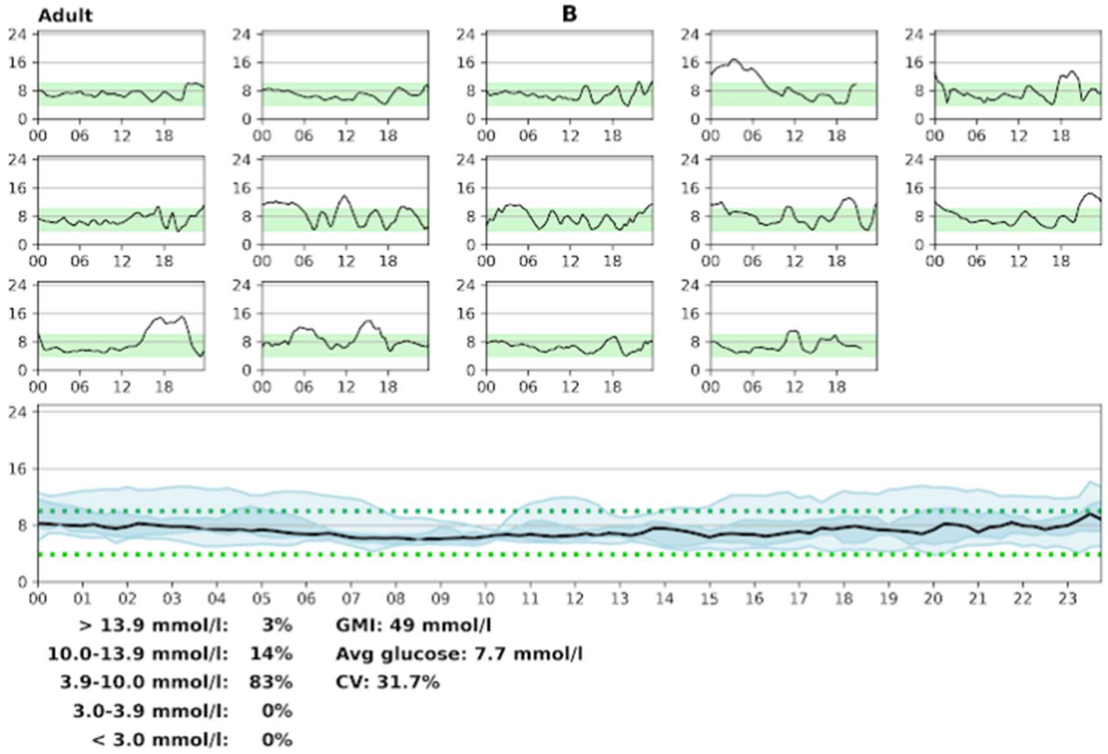

○ Set A

○ Set B

Set A: Single score of 32 (variability)

Set B: Single score of 90 (hyperglycemia)

4.4 *

Which of these two 14-days periods is most alarming?

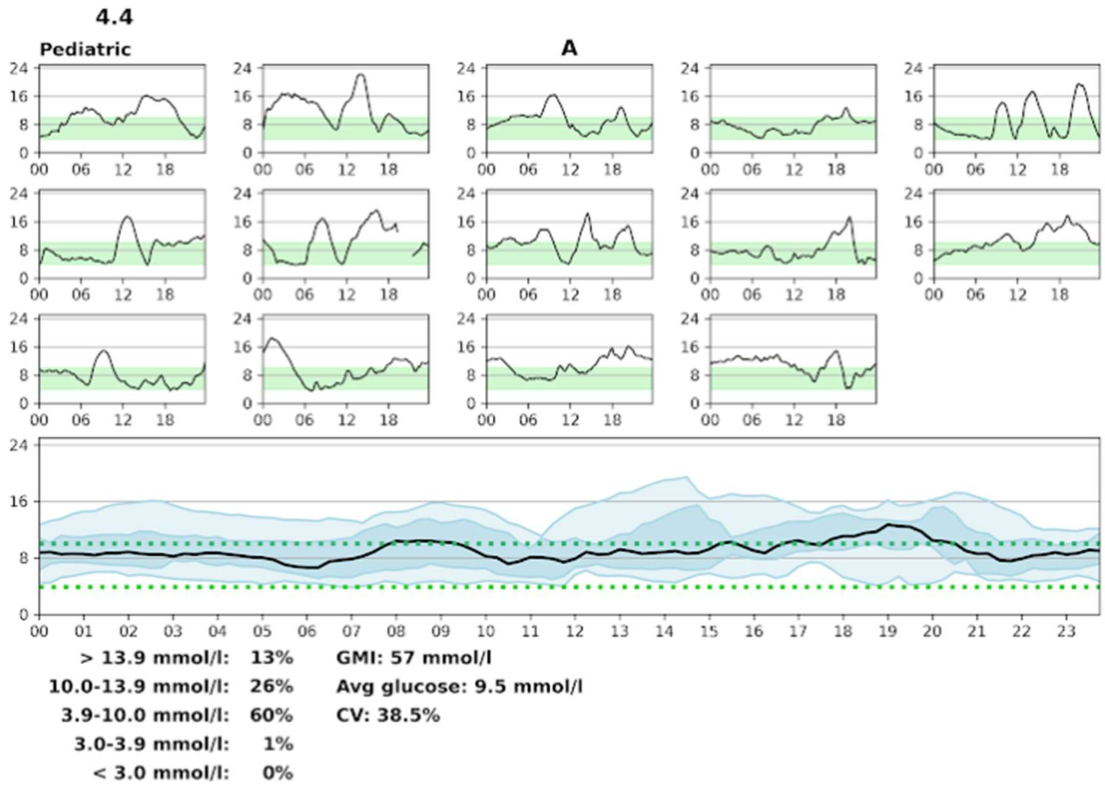

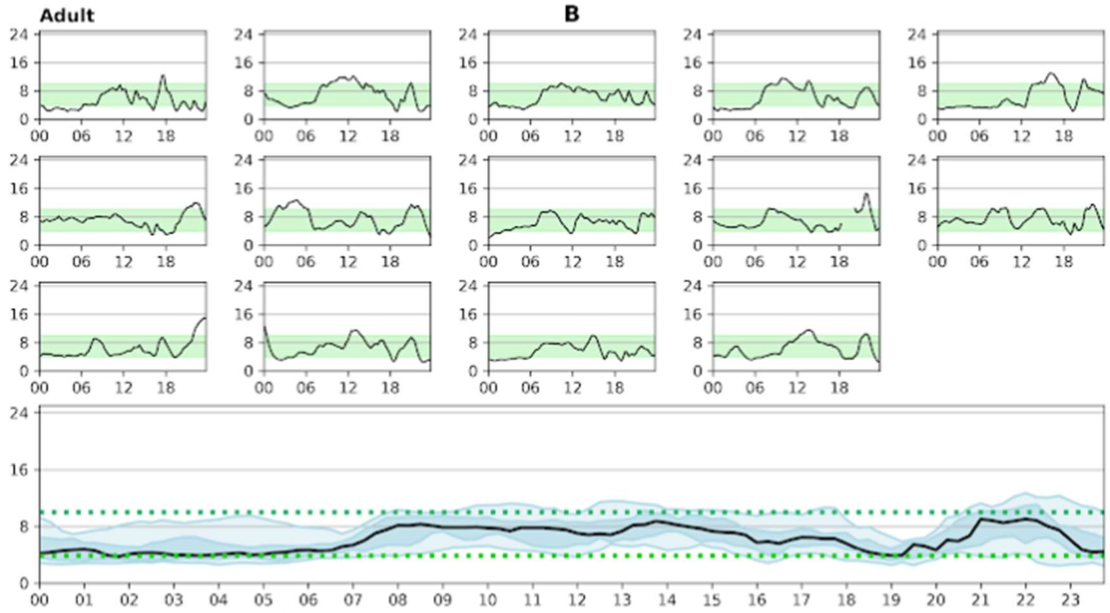

> 13.9 mmol/l:	0%	GMI: 43 mmol/l	
10.0-13.9 mmol/l:	10%	Avg glucose: 6.4 mmol/l	
3.9-10.0 mmol/l:	70%	CV: 40.0%	
3.0-3.9 mmol/l:	14%		
< 3.0 mmol/l:	5%		

- ◯ Set A
- ◯ Set B

Set A: Single score of 70 (hyperglycemia)

Set B: Single score of 31 (hypoglycemia)

4.7 *

Which of these two 14-days periods is most alarming?

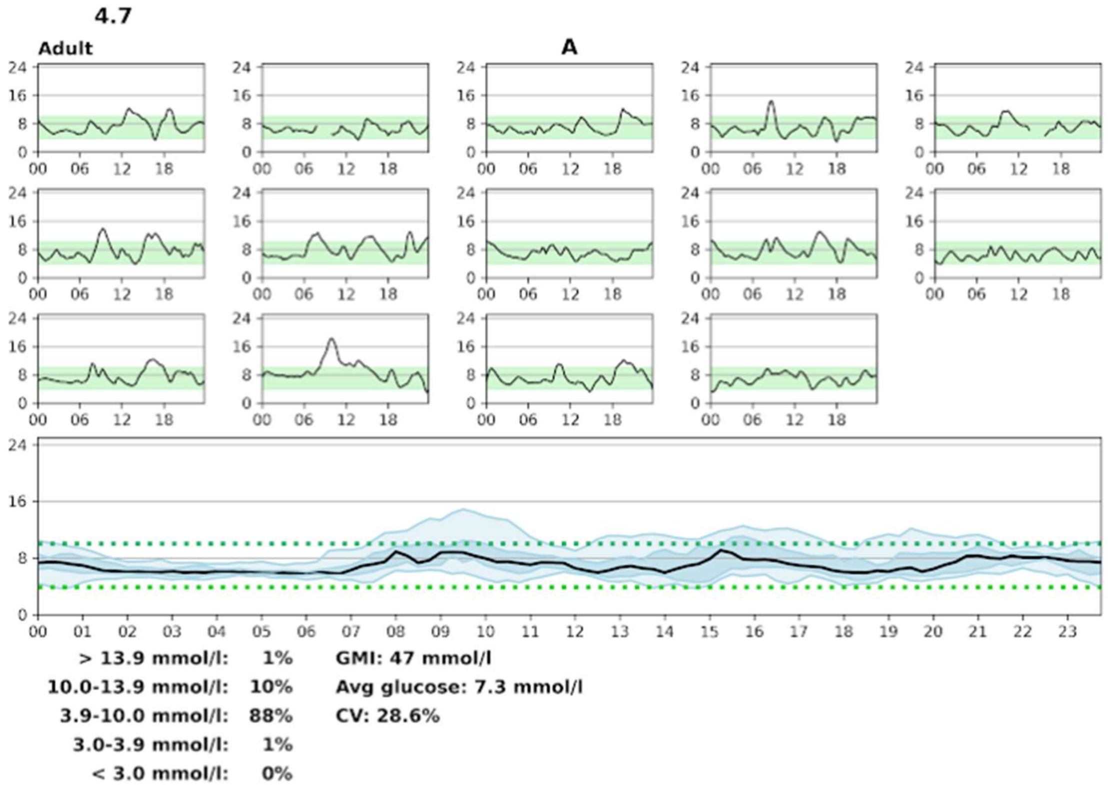

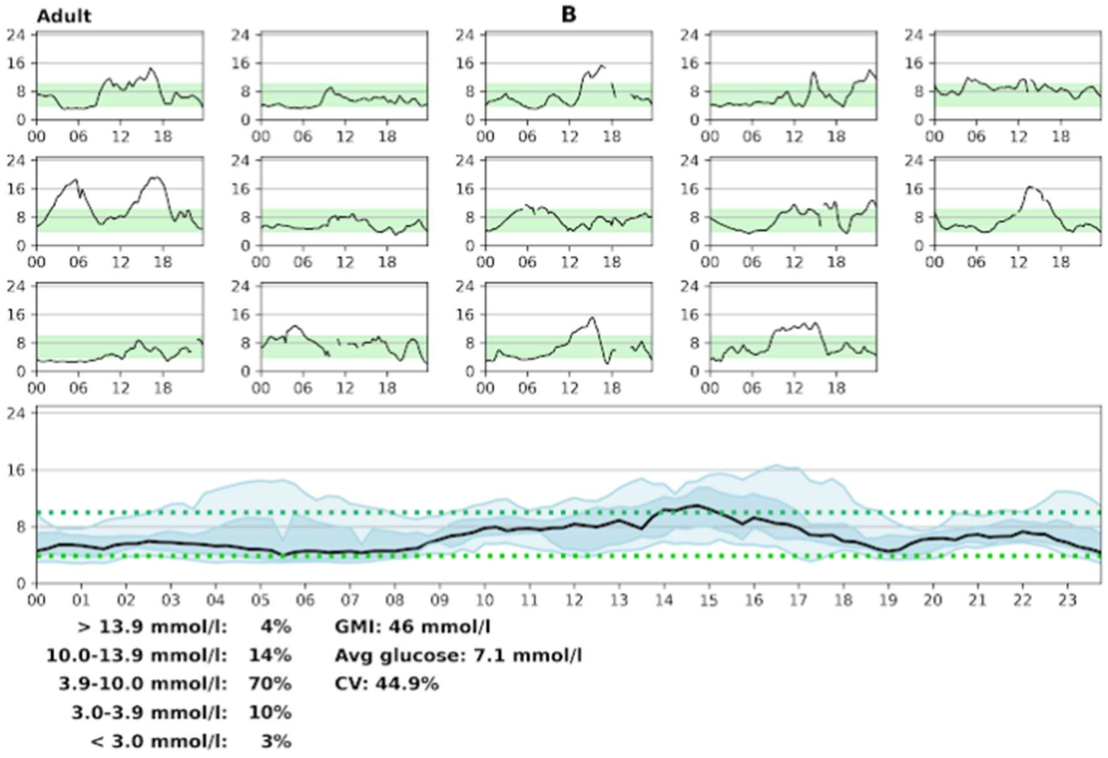

- Set A
- Set B

Set A: Single score of 86 (variability)

Set B: Single score of 51 (hypoglycemia)

Test 5. 14-days CGM curves ranking

5.1 *

Which of these two 14-days periods is most alarming?

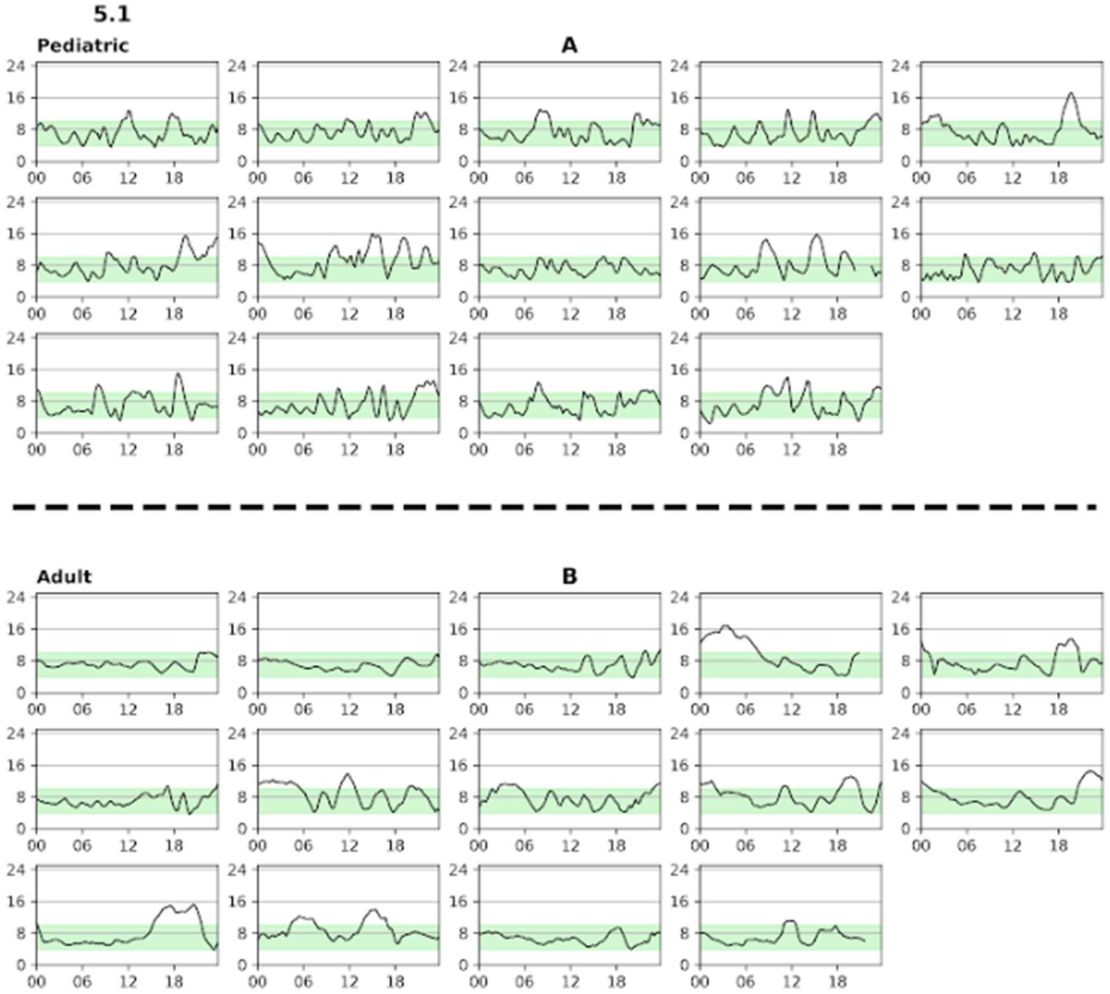

- ◯ Set A
- ◯ Set B

Set A: Single score of 32 (variability)

Set B: Single score of 90 (hyperglycemia)

5.4

Which of these two 14-days periods is most alarming? *

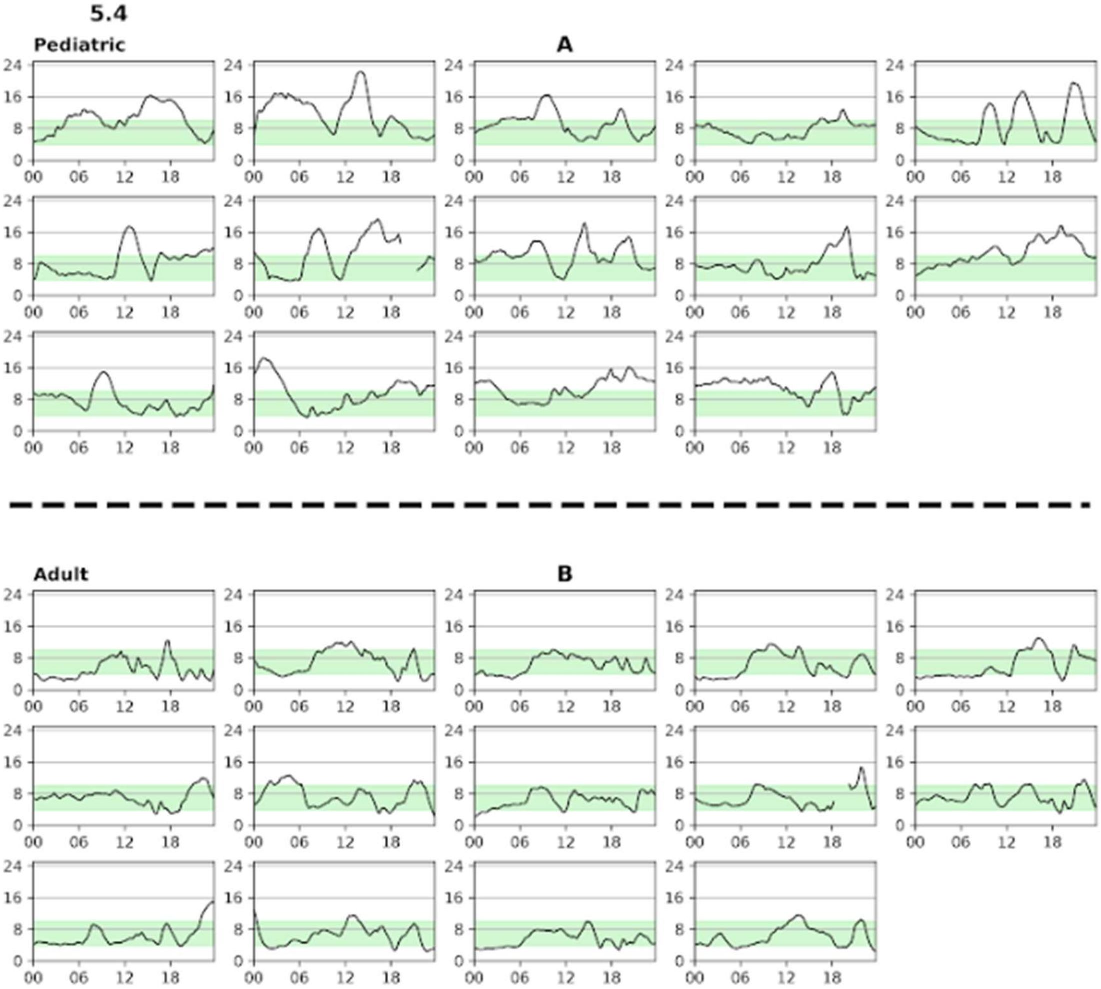

○ Set A

○ Set B

Set A: Single score of 70 (hyperglycemia)

Set B: Single score of 31 (hypoglycemia)

5.7 *

Which of these two 14-days periods is most alarming?

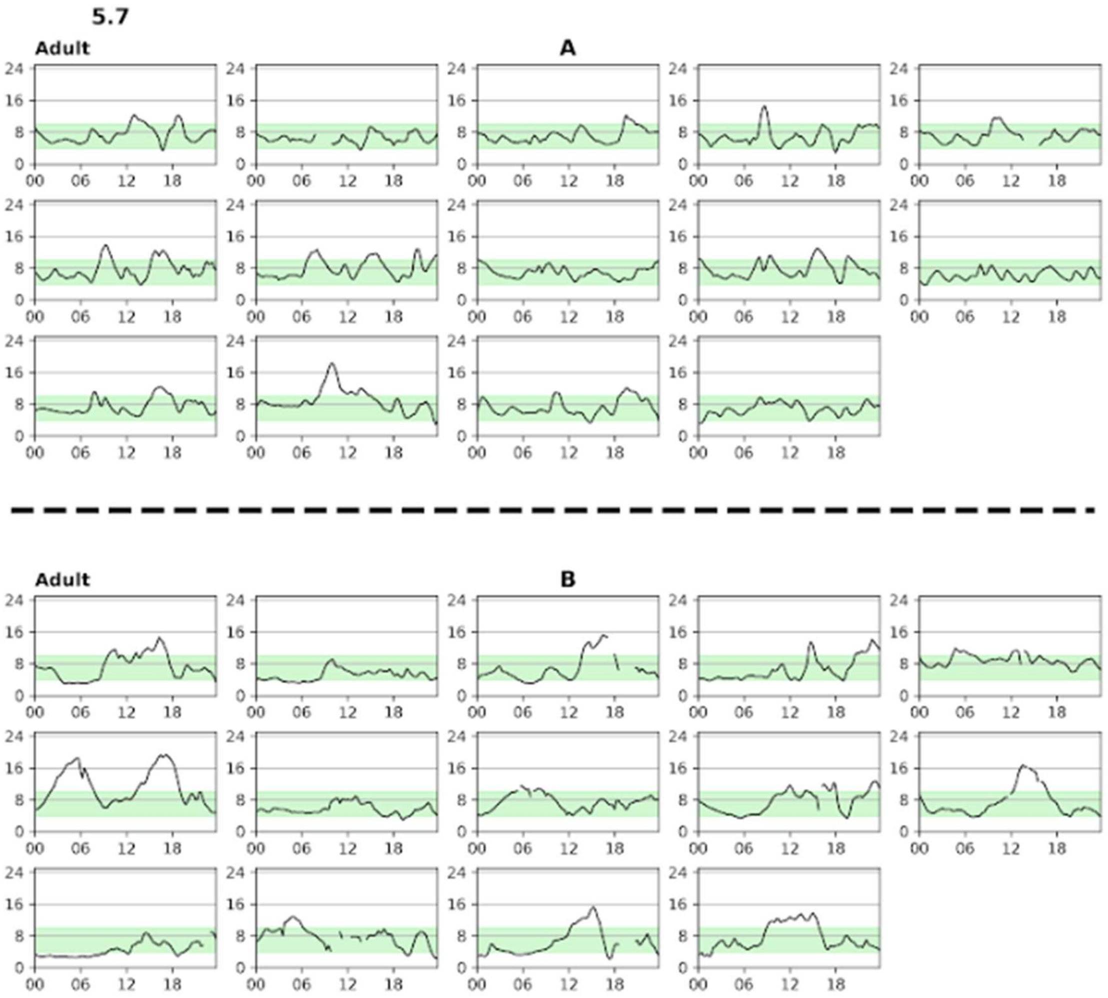

○ Set A

○ Set B

Set A: Single score of 86 (variability)

Set B: Single score of 51 (hypoglycemia)
