# Supplementary material for: Development of a three-dimensional scoring model for the assessment of continuous glucose monitoring data in type 1 diabetes
Source: BMJ Open Diabetes Res Care. 2024 Sep 5;12(4):e004350. doi: 10.1136/bmjdrc-2024-004350 (PMC11381645; doi:10.1136/bmjdrc-2024-004350)

Supplementary Figure 1 – The impact of missing data on the scoring model

Differences in score between the "true score" and the score when simulating the impact of missing data.
A) The impact of randomly dropping the data availability in steps of 10%.
B) The impact of gradually decreasing the data availability with whole days of data.
A positive difference in the histogram represents a falsely higher score.

A

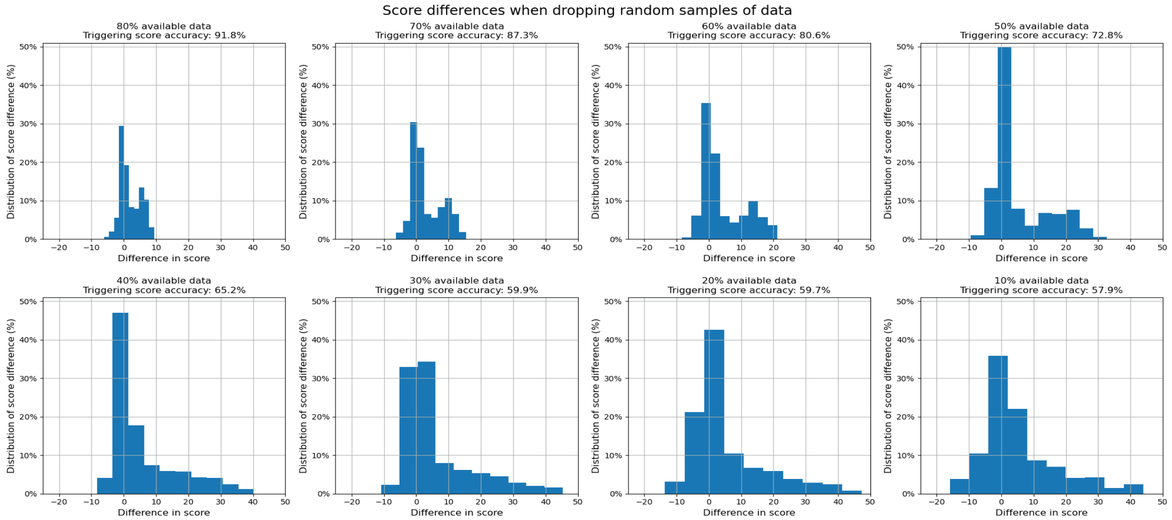

B

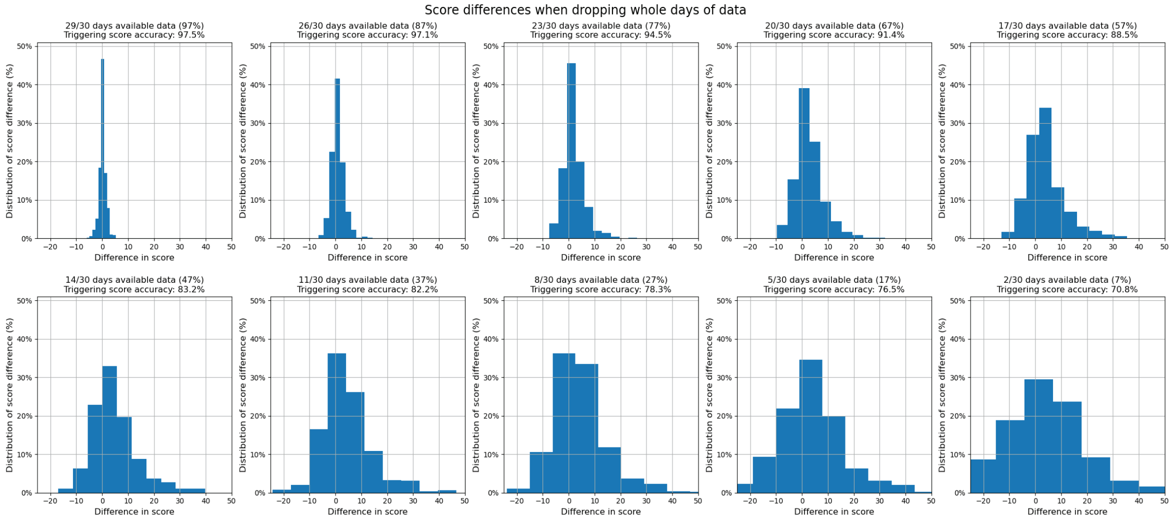

Supplement: online supplemental figure 1 [file bmjdrc-12-4-s002.pdf]
