# Supplementary material for: Development of a three-dimensional scoring model for the assessment of continuous glucose monitoring data in type 1 diabetes
Source: BMJ Open Diabetes Res Care. 2024 Sep 5;12(4):e004350. doi: 10.1136/bmjdrc-2024-004350 (PMC11381645; doi:10.1136/bmjdrc-2024-004350)

Supplementary Figure 2 – Modeling of score and AGP-metrics

Score from all available 14-day time-periods from the patient cohort and the corresponding linear regression polynomial model for Time in Range, Time below Range, Time severe below Range, Time above Range, Time severe above Range, coefficient of variation (CV%) and GLucose Management Indicator (GMI).

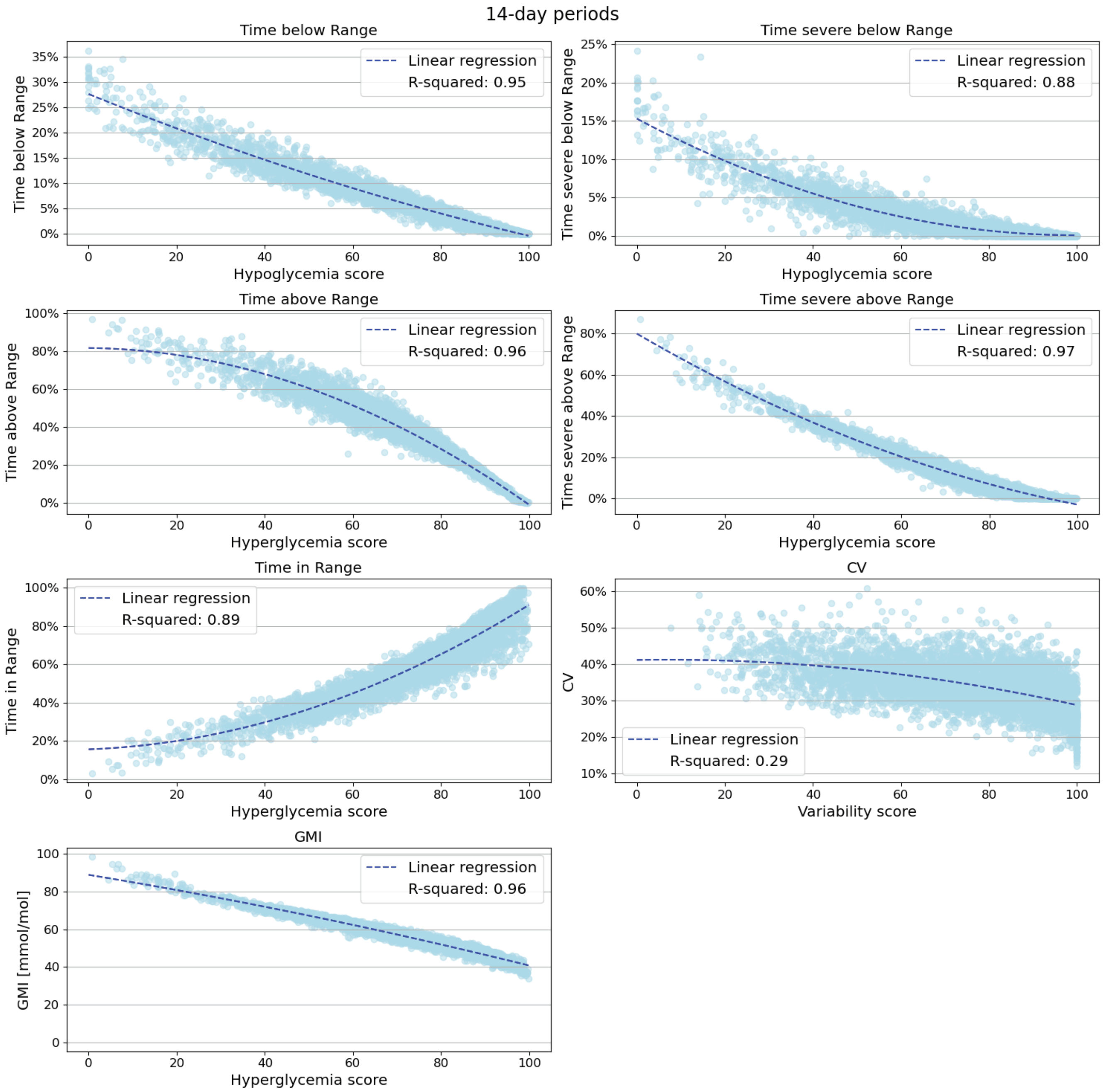

Supplement: online supplemental figure 2 [file bmjdrc-12-4-s003.pdf]
