# Supplementary material for: Development of a three-dimensional scoring model for the assessment of continuous glucose monitoring data in type 1 diabetes
Source: BMJ Open Diabetes Res Care. 2024 Sep 5;12(4):e004350. doi: 10.1136/bmjdrc-2024-004350 (PMC11381645; doi:10.1136/bmjdrc-2024-004350)
Supplement: online supplemental table 1 [file bmjdrc-12-4-s004.pdf]

Supplementary Table 1. AGP metrics and their target values, according to consensus guidelines, and the corresponding dimension of score used in the linear regressions.

**AGP metric**	**Clinical target**	**Relevant dimension of score**
Time below Range %<br>(< 3.9 mmol/L)	< 4	Hypoglycemia
Time severe below Range %<br>(< 3.0 mmol/L)	< 1	Hypoglycemia
Time above Range %<br>(> 10 mmol/L)	< 25	Hyperglycemia
Time severe above Range %<br>(> 13.9 mmol/L)	< 5	Hyperglycemia
Time in Range %<br>(3.9 – 10 mmol/L)	> 70	Hyperglycemia
Coefficient of variation %<br>(CV%)	≤ 36	Variability
Glucose management<br>indicator (GMI, mmol/mol)	≤ 52 (Adults)<br>≤ 48 (Pediatric)	Hyperglycemia
