# Supplementary material for: Development of a three-dimensional scoring model for the assessment of continuous glucose monitoring data in type 1 diabetes
Source: BMJ Open Diabetes Res Care. 2024 Sep 5;12(4):e004350. doi: 10.1136/bmjdrc-2024-004350 (PMC11381645; doi:10.1136/bmjdrc-2024-004350)
Supplement: online supplemental table 2 [file bmjdrc-12-4-s005.pdf]

Supplementary Table 2. AGP-metrics derived from the linear regression models for each segment of score.

**Targets within each segment of score**	**Green**	**Yellow**	**Orange**	**Red**	**Dark red**
Score	≥ 80	60-79	40-59	20-39	<20
Time in Range % (3.9 – 10 mmol/l)	≥ 65	65 – 45	30 – 45	20 – 30	< 20
Time below range % (< 3.9 mmol/L)	≤ 4	4 – 9	9 – 15	15 – 21	> 21
Time severe below range % (< 3.0 mmol/L)	≤ 1	1 – 2	2 – 6	6 – 10	> 10
Time above range % (> 10 mmol/L)	≤ 28	28 – 51	51 – 68	68 – 78	> 78
Time severe above Range % (> 13.9 mmol/L)	≤ 7	7 – 20	20 – 37	37 – 57	> 57
Coefficient of variation (CV%)	≤ 34	34 – 37	37 – 40	40 – 41	> 41
Glucose management indicator (GMI, mmol/mol)	≤ 52	52 – 62	62 – 72	72 – 81	> 81
